# Genetic validation of *Leishmania* genes essential for amastigote survival *in vivo* using *N*-myristoyltransferase as a model

**DOI:** 10.1186/s13071-020-3999-1

**Published:** 2020-03-14

**Authors:** Daniel Paape, Catriona T. Prendergast, Helen P. Price, Johannes S. P. Doehl, Deborah F. Smith

**Affiliations:** 1 Centre for Immunology and Infection, Department of Biology, University of York, York, YO10 5DD UK; 2 Present Address: Wellcome Centre for Molecular Parasitology, Institute of Infection, Immunity and Inflammation, University of Glasgow, Glasgow, G12 8TA UK; 3 Present Address: Institute of Infection, Immunity and Inflammation, University of Glasgow, Glasgow, G12 8TA UK; 4 Present Address: Centre for Applied Entomology and Parasitology, School of Life Sciences, Keele University, Newcastle-under-Lyme, ST5 5BG UK

**Keywords:** *Leishmania*, Plasmid shuffle, Mouse infection, Therapeutic target validation

## Abstract

**Background:**

Proving that specific genes are essential for the intracellular viability of *Leishmania* parasites within macrophages remains a challenge for the identification of suitable targets for drug development. This is especially evident in the absence of a robust inducible expression system or functioning RNAi machinery that works in all *Leishmania* species. Currently, if a target gene of interest in extracellular parasites can only be deleted from its genomic locus in the presence of ectopic expression from a wild type copy, it is assumed that this gene will also be essential for viability in disease-promoting intracellular parasites. However, functional essentiality must be proven independently in both life-cycle stages for robust validation of the gene of interest as a putative target for chemical intervention.

**Methods:**

Here, we have used plasmid shuffle methods *in vivo* to provide supportive genetic evidence that *N*-myristoyltransferase (NMT) is essential for *Leishmania* viability throughout the parasite life-cycle. Following confirmation of NMT essentiality in vector-transmitted promastigotes, a range of mutant parasites were used to infect mice prior to negative selection pressure to test the hypothesis that NMT is also essential for parasite viability in an established infection.

**Results:**

Ectopically-expressed *NMT* was only dispensable under negative selection in the presence of another copy. Total parasite burdens in animals subjected to negative selection were comparable to control groups only if an additional *NMT* copy, not affected by the negative selection, was expressed.

**Conclusions:**

*NMT* is an essential gene in all parasite life-cycle stages, confirming its role as a genetically-validated target for drug development.
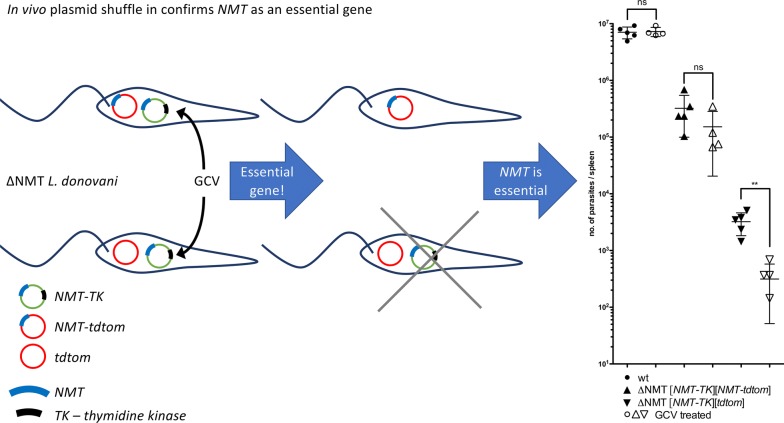

## Background

The kinetoplast parasites, *Leishmania* spp., alternate between two distinct life-cycle stages: the flagellated and motile extracellular promastigotes, and the immotile intracellular amastigotes bearing their rudimentary flagellum [1, 2]. Extracellular promastigotes develop within the female blood-feeding sand fly vector prior to transmission to the mammalian host during a blood meal. Following uptake by professional phagocytes (e.g. macrophages, dendritic cells), promastigotes then differentiate into intracellular amastigotes. These infections result in a spectrum of diseases termed the leishmaniases, the most severe forms of which are fatal in man [3, 4]. Currently, there are only a handful of licensed drugs available to treat these infections, with most having severe side effects while being difficult to administer and often requiring patient hospitalization [5]. In addition, resistance has developed in the field against some current drugs and all show varying degrees of efficacy against the differing species of infecting *Leishmania* parasite [6].

Despite some advances in drug re-purposing [6, 7], novel drug development for the leishmaniases has not been a priority for the pharmaceutical industry, even though there is an urgent need for new approaches to the treatment of these deadly infections. Current efforts are focused on the identification of compounds that target and kill intracellular amastigotes [8, 9]. Two approaches are generally available for such screening programmes: phenotypic screening or screening against a known drug target [10, 11]. Phenotypic screening has the advantage of identifying selective “cidal” compounds from high throughput screens of intracellular amastigotes, potentially identifying previously unknown/unexplored therapeutic pathways. A disadvantage of this approach, however, is that modes of action of specific compounds may be difficult to determine, although with the advent of metabolomic and chemical proteomic approaches, target deconvolution should be possible. In addition, compound optimisation is difficult when the parasite target is unknown. These challenges are obviated in target-based screening, where both the target identity and its mode of action can be studied in detail, leading to compound optimisation guided by structural constraints and definition of a structure activity relationship [12, 13].

Given the potential advantages of using target-based screening approaches and the necessity for testing against intracellular amastigotes it is crucial that the target in question is essential for parasite viability within the intracellular environment. The target has to be a known gene coding for an essential protein required for parasite viability in the host. At present, any target gene of interest is assumed to be essential for viability if it can only be deleted from its genomic locus when its product is expressed ectopically in the extracellular promastigote stage of the parasite life-cycle [14, 15]. In practice, these gene replacements can be technically complex, often requiring use of more than two selectable markers, dependent on the location of the gene of interest, to account for the variable chromosomal ploidy of *Leishmania* species [16]. The data generated from such genetically manipulated promastigote strains are then often correlated with gene function in intracellular amastigotes, despite the lack of phenotypic information derived from genuine gene knockouts generated in amastigotes. Generating such information is not only important for the robust identification of new drug targets in intracellular parasites, but will also inform our understanding of parasite biology, ensuring, for example, that there are no appropriate biochemical “escape” pathways to facilitate amastigote survival rendering drug treatment futile and to predict adaptation of the parasites to inhibition.

To date, and to our knowledge, no gene essential for promastigote viability has also been definitively shown by genetic manipulation to be essential for viability in the *Leishmania* amastigote. This is principally due to the lack of a robust inducible expression system [17] and a functioning RNAi machinery [18] that works in all *Leishmania* species.

*N*-myristoyltransferase (NMT) catalyses the covalent attachment of a myristate moiety to the N-terminal glycine of selected eukaryotic target proteins, with 60 proteins predicted to be myristoylated (reviewed in [19, 20]). Experimentally 30 proteins were confirmed with high and 18 with lower confidence to be a substrate of NMT [21]. Gene knockout by homologous recombination has been used to demonstrate that NMT is an essential enzyme in extracellular promastigotes of *Leishmania* [22, 23] as well as in both insect and mammalian extracellular stages of *Trypanosoma brucei* [23]. As a consequence, NMT is being exploited as a potential target for chemotherapeutic intervention across the leishmaniases, with high-throughput screening leading to the identification of first generation compounds with specificity and sensitivity against *Leishmania* promastigotes [21, 24–26].

Plasmid shuffle is based on the concept that a plasmid carrying an essential gene is only dispensable in the presence of another functional copy of the gene or if necessary metabolic products are present compensating for the lack thereof [27]. It has already been applied to investigate gene function in *Leishmania*, but only in promastigote stages [28–31]. Methodologically, the gene of interest is expressed ectopically from a plasmid before it is deleted from its genomic locus. To direct the plasmid shuffle in *Leishmania*, this plasmid also encodes for thymidine kinase (TK) a negative selective marker, to force the death of the parasite or the loss of this plasmid upon negative selection with ganciclovir (GCV), a nucleoside analogue. TK phosphorylation occurs and the resulting nucleotide can be used by the parasites, interfering with strand elongation during DNA replication. If parasites die or the plasmid is only dispensable in the presence of another functional gene copy upon negative selection, then the gene can be regarded to be essential. Here, we have applied plasmid shuffle methodology to provide supportive evidence that NMT is essential in both life-cycle stages of *Leishmania* parasites, thereby confirming its potential as a fully validated drug target in these species.

## Methods

### *Leishmania in vitro* culture

Promastigotes of *L. donovani* MHOM/ET/67/L28 (LV9 strain) were grown at 26 °C in RPMI 1640 medium (Gibco, Paisley, UK) supplemented with 20% heat-inactivated FBS, 100 µM adenine, 20 mM 2-[N-morpholino] ethanesulphonic acid (pH 5.5), 5 µM hemin, 3 µM biopterin, 1 µM biotin (all Sigma-Aldrich, Irvine, UK), 100 U/ml penicillin and 100 μg/ml streptomycin (GIBCO, UK).

### *In vivo* infections

BALB/c mice were obtained from Charles River (UK) and infected with 3 × 10^7^*L. donovani* stationary phase promastigotes, in 200 μl of RPMI 1640 (GIBCO), intravenously (i.v.) *via* the tail vein. Ganciclovir (GCV, Invivogen, Toulouse, France) was dissolved in H_2_O pH 12 at 10 mg/ml and then diluted to 1.25 mg/ml in 0.1 M HEPES pH 7.4. Solvent control was prepared in an identical manner but without the GCV. Mice were treated with 3 mg/kg b.i.d (morning and evening (10–12 h apart) by intraperitoneal injection. Control mice were injected with the respective volume of solvent solution.

### Generation of NMT-expression plasmids

*NMT* was amplified from genomic DNA (forward primer: 5′-ACG AGA TCT ATG TCT CGC AAT CCA TCG AAC TC-3′; reverse primer: 5′-GCT AGA TCT CTA CAA CAT CAC CAA GGC AAC C-3′) and cloned into the *Bgl*II restriction site (restriction sites are underlined throughout) of pXNG4-*SAT* (also encoding TK and kindly provided by S. Beverley, Washington University, St Louis, USA and described elsewhere [31]) to obtain pXNG4-*SAT*-*NMT*. The plasmids pX-NEO-tdtom and pX-NEO-NMT-tdtom (tdtom denotes tandem tomato fluorescent protein - *tdTomato*) were generated by amplifying LamDH intergenic region (IR) (forward primer: 5′-ACC TCT AGA ACA TCG ATT GTG GAA GCA CAA AGC GCA C-3′; reverse primer: 5′-AAT TCT AGA CAT ATG CAA GCT GAT CCA GAG GAC GTG-3′) and Lam NAGT IR (forward primer: 5′-TCA GGA TCC GAT CCA GTA GTG CCA ATA GAG-3′; reverse primer: 5′-ATT GCG GCC GCT CAT GTT TGA CAG CTT ATC ATC-3′) from pXNG4-SAT and cloning into the *Xba*I and *BamH*I/*Not*I restriction sites, respectively, of the pX expression vector [32]. Tdtom was generated by amplification from pSSU-tomato [33]; forward primer: 5′-GAG CAT ATG GTG AGC AAG GGC GAG GAG-3′; reverse primer: 5′-ATA GCG GCC GCA CGC GCC GGG CAT CGC TGC-3′) and cloned into the previously introduced *Nde*I site (reverse primer Lam NAGT IR) and *Not*I site; *NMT* was amplified from genomic DNA (forward primer: 5′-GAA CCC GGG ATG TCT CGC AAT CCA TCG AAC TC-3′; reverse primer: 5′-CCA TCT AGA CTA CAA CAT CAC CAA GGC AAC C-3′) and cloned into the *Xma*I and *Xba*I sites to obtain pX-NEO-NMT-tdtom.

### Transfected *Leishmania* lines

For generation of conditional double *NMT* allele replacement mutants, *L. donovani* NMT^HYG/+^ promastigotes [22] were transfected with pXNG4-SAT-NMT. Replacement of the second *NMT* allele was achieved by targeted gene replacement as described [22]. One confirmed genomic homozygous *NMT* knockout carrying the pXNG4-SAT-NMT plasmid line was then used for transfection with pX-NEO-tdtom or pX-NEO-NMT-tdtom.

Transfections were performed sequentially with 5 μg purified DNA by nucleofection (using the Human T Cell Nucleofector Kit, Lonza, Basel, Switzerland) of 2 × 10^7^ mid-log phase promastigotes. Mutants were selected on RPMI medium/1% agar plates with respective antibiotics (32 μg/ml hygromycin B; 40 μg/ml G418; 80 μg/ml puromycin; 100 μg/ml nourseothricin). PCR screening to confirm the correct targeting of the 2nd allele was performed with the following forward primers: PACint (5′-ACC TGG TGC ATG ACC CGC AAG-3′), HYG (5′-CCT GAA CTC ACC GCG ACG TC-3′) and LdNMT (5′-CTA TGC CCA CCG AGC TAC ATC C-3′) together with the reverse primer: Syntaxin Rev (5′-GCC AGC TGC GTC AAA CGC AT-3′).

### Intracellular *in vitro* analysis

Bone marrow was extracted from 6–8 week-old female BALB/c mice and differentiated to macrophages with L929 conditioned medium. Alternatively, THP-1 cells were transformed to macrophages with phorbol 12-myristate 13-acetate (PMA) or retinoic acid as described in [34] or [35], respectively. Macrophages were infected with a MOI of 10 with stationary promastigotes. Upon transformation to amastigotes, in our hands it took 72 h, the cells were either treated with 50 μg/ml GCV or solvent. At the appropriate time points, PMA stimulated cell were lifted of the well bottom upon two washes with PBS with a cell scraper and then collected as described in [35]. Retinoic acid-stimulated cells were harvested as described in [35]. Cells were then subjected to flow cytometric or microscopic analysis.

### Flow cytometry

GFP and tdtom expression was assessed in live promastigotes suspended in PBS by flow cytometry. Cells were analysed on a DAKO CyAn ADP analyser (DakoCytomation, Ely, UK) and data were analysed with FlowJo v10.0.6 (TreeStar Inc., Ashland, OR, USA).

### Parasite burdens

Splenic parasite burdens were determined by limiting dilution assay as described [36]. Spleens were dounce-homogenized and then plated out as described, in the presence/absence of the following concentrations of selection antibiotics as appropriate: hygromycin (32 μg/ml); nourseothricin (100 μg/ml); puromycin (80 μg/ml); G418 (40 μg/ml).

### Statistics

Analysis of statistically significant differences between groups were performed by Mann–Whitney test using GraphPad Prism v8 (GraphPad Software Inc., San Diego, CA, USA)

## Results

### Generation and characterisation of conditional genomic *NMT* allele deletions

**Fig. 1:**
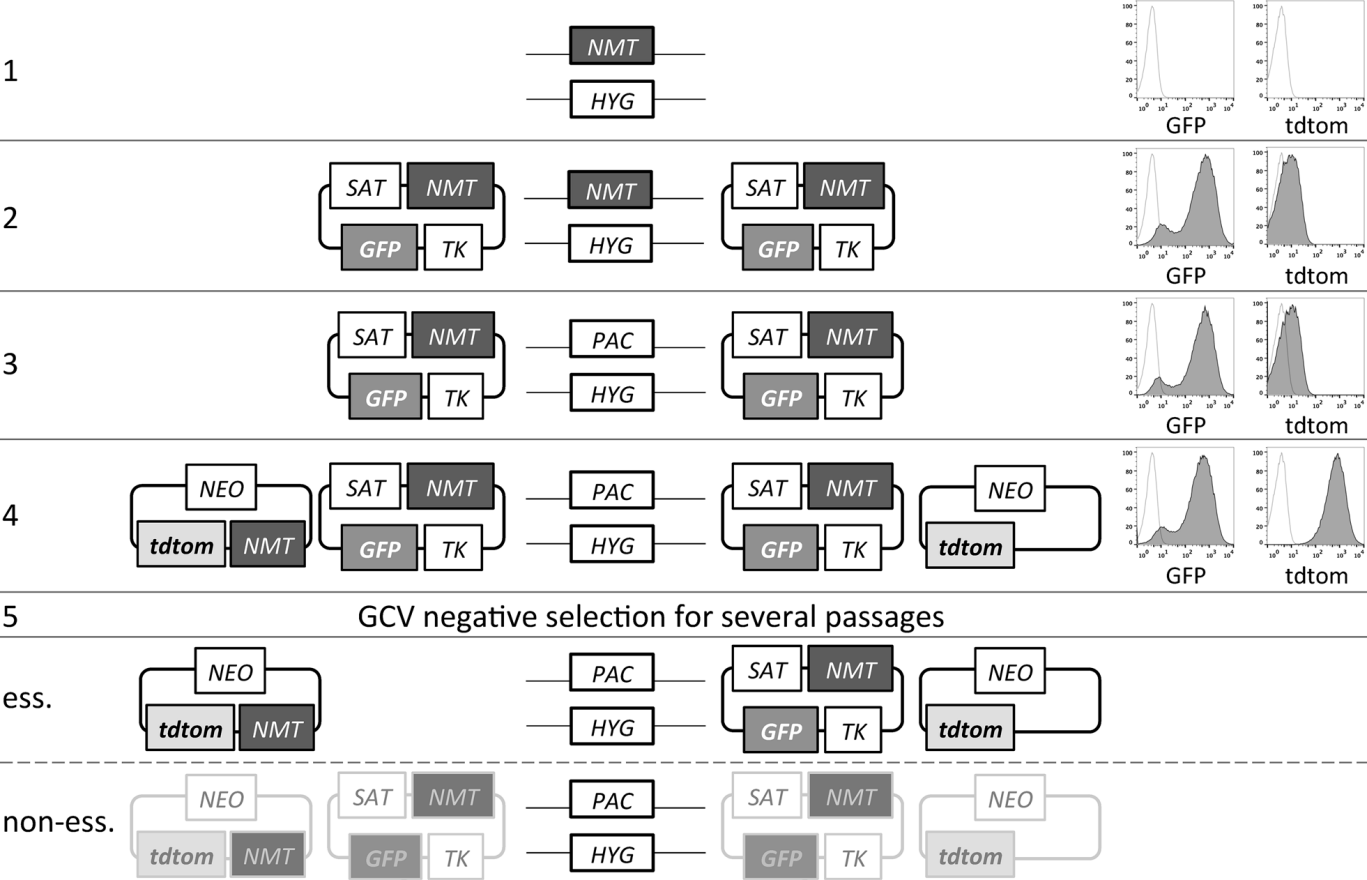
Overview of plasmid shuffle experimental procedure. Generation of conditional *L. donovani NMT* null mutants to demonstrate *NMT* essentiality by plasmid shuffle. Heterozygous *NMT*^*HYG*/+^ promastigotes (**1**) were episomally transfected with pXNG4-*NMT-TK* carrying *GFP* and *TK* (**2**), before targeting the 2nd *NMT* allele for replacement with *PAC*, creating *NMT*^*PAC*/*HYG*^ (**3**). Individual clones were then transfected with an episomal plasmid coding for tdtom only or for *NMT* and tdtom (NMT-tdtom; **4**). Ectopic expression of *NMT* by only one (pXNG4-*NMT-TK*) of the two plasmids (right hand side scenario) or both (left hand side scenario) will allow genetic validation of its essentiality by negative selection with ganciclovir (GCV, **5**). If *NMT* is an essential gene, mutants will grow, after several passages of negative selection that are only tdtom+, when parasites were transfected with pXNG4-*NMT-TK*/NMT-tdtom, or GFP+ and tdtom+, when transfected with pXNG4-*NMT-TK*/tdtom, respectively. The latter may not grow but rather die due to the negative selection. If *NMT* is non-essential for viability, retention of plasmids during cytokinesis will be subject to a random distribution and tdtom+ and/or GFP+ mutants could arise in both conditional complementation settings or the plasmids will not be retained at all. Histograms shown on the right in **1–4** depict representative fluorescent properties for GFP and tdtom of the heterozygous *NMT*^*HYG*/+^ parasites (1- starting population) and mutants generated by episomal plasmid transfection (**2–4**, grey filled curve, no fill shows starting population). *Abbreviations*: *NMT*, *N*-myristoyltransferase; *SAT*, streptothricin acetyltransferase; *HYG*, hygromycin phosphotransferase; *PAC*, puromycin *N*-acetyltransferase; *NEO*, neomycin phosphotransferase; GFP, green fluorescent protein; tdtom, tandem tomato fluorescent protein; TK, thymidine kinase from Herpes simplex virus; ess, essential

**Fig. 2:**
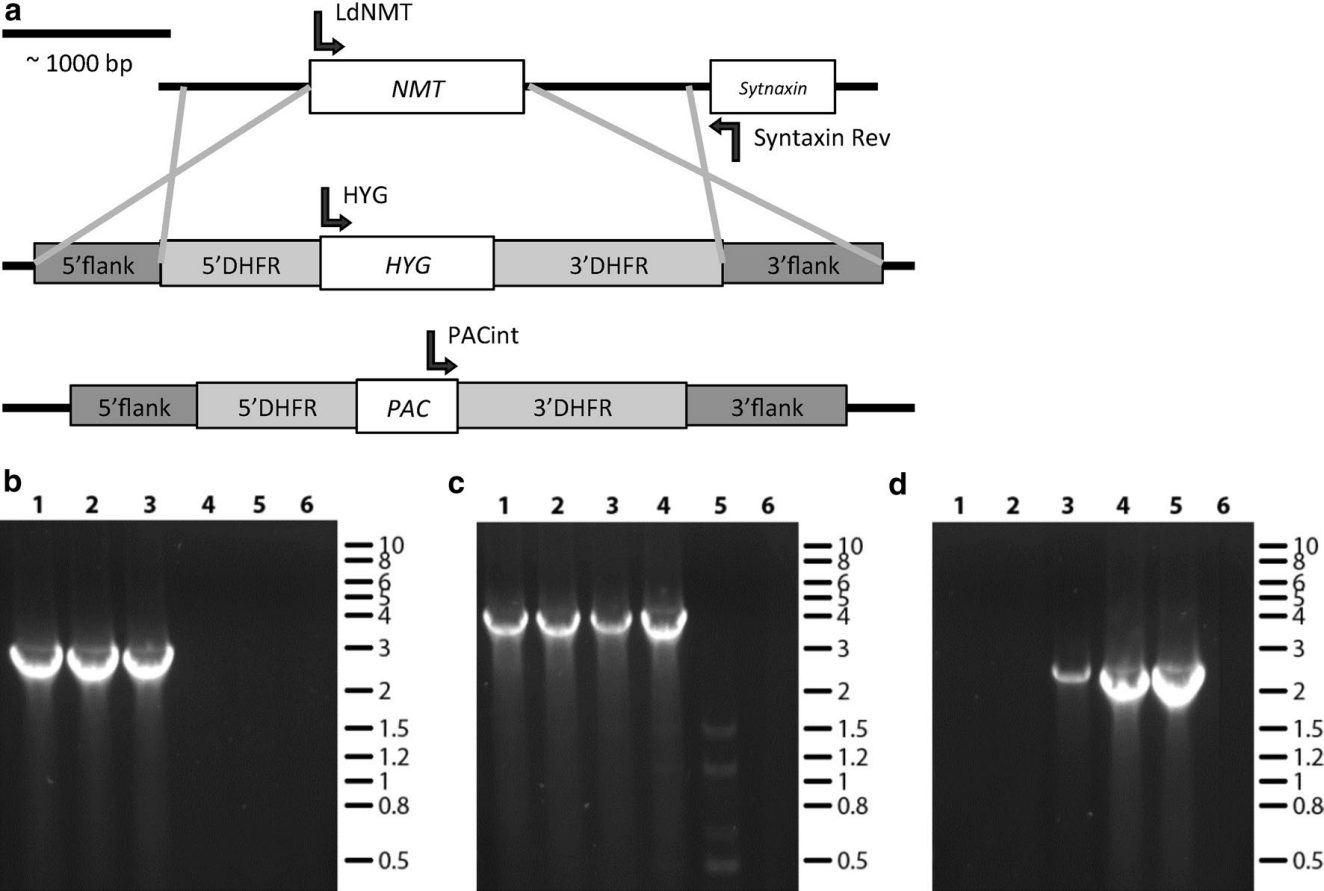
PCR analysis of *NMT* complemented double replacement *HYG*/*PAC* parasites. **a** Diagram of *NMT* locus and targeted single alleles containing replacement by either the *HYG* or *PAC* genes. 5′flank and 3′flank boxes represent *NMT* flanking regions used for gene targeting. 5′DHFR and 3′DHFR boxes represent 5′ and 3′ dihydrofolate reductase flanking regions. Arrows show positions of primers used in PCR analysis of clones in **b–d**. Lane 1: LV9 *NMT* ^*HYG*/*PAC*^ [NMT-TK]; Lane 2: LV9 *NMT*^*HYG*/*PAC*^ [NMT-TK] clone 12; Lane 3: LV9 *NMT*^*HYG*/*PAC*^ [NMT-TK] clone 18; Lane 4: LV9 *NMT*^*HYG*/+^; Lane 5: LV9 wild type; Lane 6: no DNA control. **b***PAC* integration, expected band 2513 bp. **c***HYG* integration, expected band 3488 bp. **d** Genomic *NMT*, expected band 2097 bp. Allelic *NMT* was only replaced completely for clone 12 while clone 18 retained the gene. Clone 12 was used to generate LV9 *NMT*^*HYG*/*PAC*^ [NMT-TK][NMT-tdtom] or LV9 *NMT*^*HYG*/*PAC*^ [NMT-TK][tdtom]

NMT had previously been shown to be essential for the viability of *L. donovani* promastigotes [22]. In the present study, in order for us to replace both genomic *NMT* alleles in these experiments, it was necessary to express an *NMT* gene copy at an ectopic locus, in this case, from one of two or two distinct plasmids expressing different reporters GFP or tdTom. The strategy for generation and use of the resulting parasite lines in these new plasmid shuffle experiments is shown in Fig. 1. Flow cytometry was used to monitor the fate of either plasmid by detecting GFP or tdtom expression. This allowed us to determine whether *NMT* is an essential or dispensable gene for parasite viability. The open reading frame of *L. donovani NMT* (ORF) was cloned into the plasmid pXNG4Sat (hereafter called NMT-TK; coding for *GFP*, *TK* and the selection marker, S-streptothricin acetyltransferase (SAT; [31]). This plasmid was transfected into *L. donovani* heterozygous for *NMT*, *NMT*^*HYG*/+^ [22]. The ectopic expression of *NMT* from the plasmid made it possible to replace the second genomic *NMT* allele (Fig. 2a-d). The resulting parasite line, LV9 Δ*NMT*^*HYG*/*PAC*^ [NMT-TK], was then transfected with a second plasmid, either encoding *tdTomato* and neomycin phosphotransferase (*NEO*) alone (hereafter called tdtom) or *tdTomato* and *NEO* plus *LdNMT* (hereafter called NMT-tdtom). The second plasmid was engineered so that the *tdTomato* was under the same regulatory control as *GFP* in the NMT-TK plasmid. The histograms depicting the fluorescent characteristics of the parasite lines generated are also shown on the right in Fig. 1. All of these parasite lines moderately overexpressed *NMT* compared to wild type (wt) levels (Additional file 1: Figure S1) but below the lethal ~5-fold limit as reported for *L. major* [23]. Table 1 summarises the three plasmids and their characteristics used here.

**Table 1:** Overview of plasmids used and their characteristics

Abbreviation used in manuscript	Fluorescence	Antibiotic gene	*N*-myristoyl-transferase present?	Thymidine kinase present?	Antibiotic used for selection
NMT-TK	GFP	Neomycin phospho-transferase	Yes	Yes	G418
NMT-tdtom	tdtom	Streptothricin acetyl-transferase	Yes	No	Nourseothricin
tdtom	tdtom	Streptothricin acetyl-transferase	No	No	Nourseothricin

### Qualitative and quantitative *in vitro* analysis

First, it was necessary to establish that the plasmid shuffle approach works in the extracellular life-cycle stage, in which NMT has already been shown to be essential for viability [23] and to monitor the effect of GCV on these promastigotes. The hypothesis was that upon negative selection, the NMT-TK plasmid could only be lost in the presence of another expressed *NMT* copy present on a second plasmid. In the absence of this second *NMT* copy, we expected the parasites to die or to maintain the NMT-TK plasmid.

**Fig. 3:**
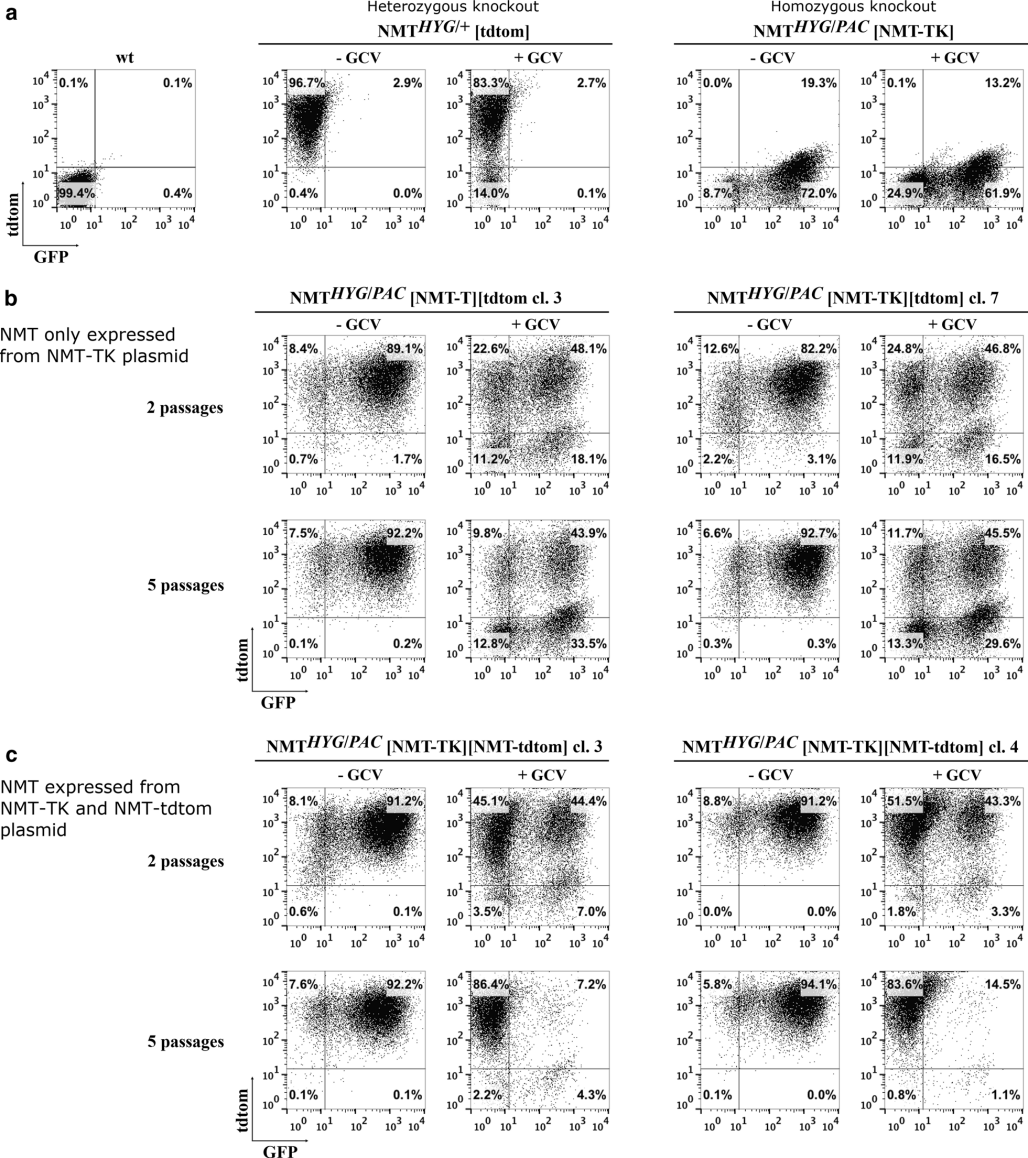
Promastigote flow cytometric assay. **a** Flow cytometric characteristics of single and double knockouts only transfected with a single plasmid in presence and absence of ganciclovir (GCV). **b** In the *NMT* double knockout parasites the plasmid pXNG4-*SAT*-*NMT* could not be lost in the absence of a second ectopic *NMT* allele. **c** The plasmid pXNG4-*SAT*-*NMT* was lost in the *NMT* double knockout parasites over several passages in the presence of a second ectopic *NMT* allele. Mutants were kept for 2 and/or 5 passages in medium containing GCV or in medium containing either hygromycin and G418 or hygromycin, puromycin and nourseothricin, respectively, at which point they where analyzed by flow cytometry. *NMT*^*HYG*/+^ [tdtom] = LV9 *NMT*^*HYG*/+^ [ tdtom] cl. 13; *NMT*^*HYG*/*PAC*^ [NMT-TK] = LV9 *NMT*^*HYG*/*PAC*^ [NMT-TK] cl. 12

No effects were observed following GCV treatment of *L. donovani NMT* single knockout (KO) promastigotes transfected with the tdtom plasmid (Fig. 3a). However, the absence of the antibiotics hygromycin and G418 in the GCV-treated samples led to the loss of this plasmid in 14% of the parasite population (Fig. 3a). Double *NMT* KO promastigotes transfected with NMT-TK did not show altered fluorescent characteristics even upon GCV treatment for 5 passages (~50 generations) and in the absence of nourseothricin to select for this plasmid. The main population remained GFP-positive as in the untreated parasites. However, the lower GFP fluorescent-positive (dim) population increased from 10.6% to 30.2% (Additional file 2: Figure S2) gate spanning ~1 log scale across the GFP dim population shown in Fig. 1, this gate is slightly shifted along the X-axis to include the majority of the GFP-dim parasites), similar to that described earlier for essential genes [28, 31] and attributed to either a low copy number of the pXNG4 plasmid [31] or reduced GFP expression, possibly due to cellular stress [28].

*Leishmania donovani* double *NMT* KO promastigotes, only expressing *NMT* from the NMT-TK plasmid, did not lose this plasmid, even after ~50 generations in the presence of 50 μg/ml GCV (Fig. 3b). The majority of these parasites were still GFP/tdtom-positive, ~45%, or purely GFP-positive, ~30%, with again a dim GFP-positive population (based on gates shown in Additional file 2: Figure S2), ~30%, of which ~14% were also tdtom-positive (Fig. 3b). A similar distribution was recorded after 2 passages (~15 generations) with GCV. There was no apparent change between the control parasites maintained for 2 or 5 passages in the appropriate antibiotics to select for the plasmids (Fig. 3b) 89.1% and 92.2% double positive, respectively.

*Leishmania donovani* promastigotes with double *NMT* KOs expressing *NMT* from the second plasmid, NMT-tdtom, did lose the NMT-TK plasmid over time when cultured in the presence of 50 μg/ml GCV, as expected. This plasmid was completely lost after ~50 generations with the parasites remaining solely tdtom-positive (Fig. 3c). In comparison, the control parasites maintained in the appropriate selecting antibiotics did not differ from the starting population (Fig. 3c). Table 2 shows the median fluorescence intensity (MFI) values of the GFP fluorescence for the GFP dim populations. Results indicate a clear and consistent skew towards higher GFP fluorescence in the groups where the NMT-TK plasmid is present in the GCV-untreated groups and in the double *NMT* KOs where NMT-TK had to be maintained. These data confirm that a copy of the *NMT* gene is essential for promastigote viability in a direct *in vitro* assay. The targeted loss of NMT-TK upon GCV treatment in parasites carrying an additional copy on *NMT* confirms that the GCV selection is functional.

**Table 2:** Median fluorescence intensity (MFI) values for the GFP signal of the GFP dim population (quadrant 1 is top left and quadrant 4 bottom left)

Genomic setting of *Leishmania* (mutant) line	Quadrant	MFI of GFP signal	
		− GCV	+ GCV
wt	Q1	na	na
	Q4	4.0	na
*NMT*^HYG/+^ [tdtom]	Q1	3.8	3.3
	Q4	na	3.9
*NMT*^HYG/PAC^ [NMT-TK]	Q1	na	na
	Q4	6.8	7.0
*NMT*^HYG/PAC^ [NMT-TK][tdtom] cl. 3	Q1	7.0	7.1
	Q4	na	7.4
*NMT*^HYG/PAC^ [NMT-TK][tdtom] cl. 7	Q1	8.2	7.2
	Q4	na	7.5
*NMT*^HYG/PAC^ [NMT-TK][NMT-tdtom] cl. 3	Q1	7.0	2.7
	Q4	na	na
*NMT*^HYG/PAC^ [NMT-TK][NMT-tdtom] cl. 4	Q1	7.3	3.7
	Q4	na	na

*Abbreviation*: na, not applicable

### Intracellular *in vitro* analysis

Next, we wanted to apply the plasmid shuffle method to test whether NMT is also essential in the intracellular life-cycle stage of *Leishmania*. At first an *in vitro* approach was pursued, with two sources of phagocytes used for infection with the promastigote mutant lines described above: murine bone marrow-derived macrophages and PMA [34] and retinoic acid [35] stimulated human THP-1 cells. Upon transformation of promastigotes to amastigotes inside these cells, macrophages were treated with GCV or solvent. However, during this transition, it was noted that the GFP fluorescent intensity had dropped considerably when compared to the starting promastigotes, such that it was not possible to discriminate between single GFP-positive cells, dim GFP-positive cells or dim GFP/tdtom-positive cells (data not shown). It was concluded from this observation that the differentiated amastigotes did not proliferate at a sufficient rate for robust detection in either of these cellular systems and the doublings needed for an efficient loss of the plasmid in a single round of *in vitro* macrophage infection are not achievable. Hence, *in vivo* whole animal experiments were initiated as an alternative approach.

### Qualitative *in vivo* analysis

**Fig. 4:**
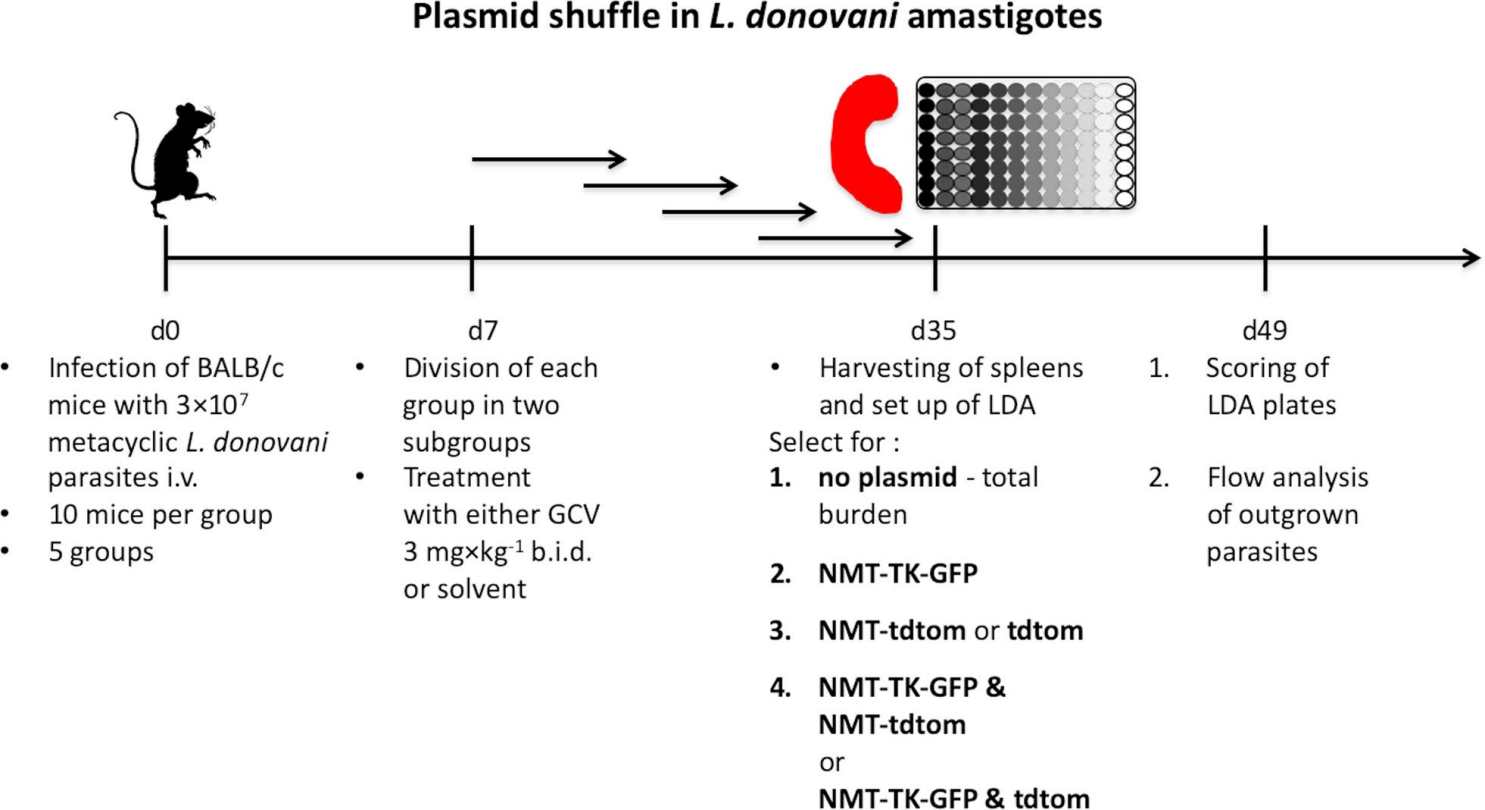
Flow chart describing the *in vivo* experimental plan. Day 35 spleens were harvested and a limiting dilution assay (LDA) set-up. Condition 1: the medium used in the LDA only contained antibiotics selecting for single *NMT* or double *NMT* knockouts, depending on the mutants initially injected, in order to determine the total parasite burden. Conditions 2–4 were set up to determine the individual parasite burden, i.e. which plasmid are the parasite still carrying: Condition 2: LDA medium contained also nourseothricin to select for the NMT-TK plasmid. Condition 3: LDA medium contained also G418 to select for the NMT-tdtom or the tdtom plasmid. Condition 4: the medium contained also nourseothricin and G418 to select either for the NMT-TK and NMT-tdtom plasmid or the NMT-TK and tdtom plasmid

**Fig. 5:**
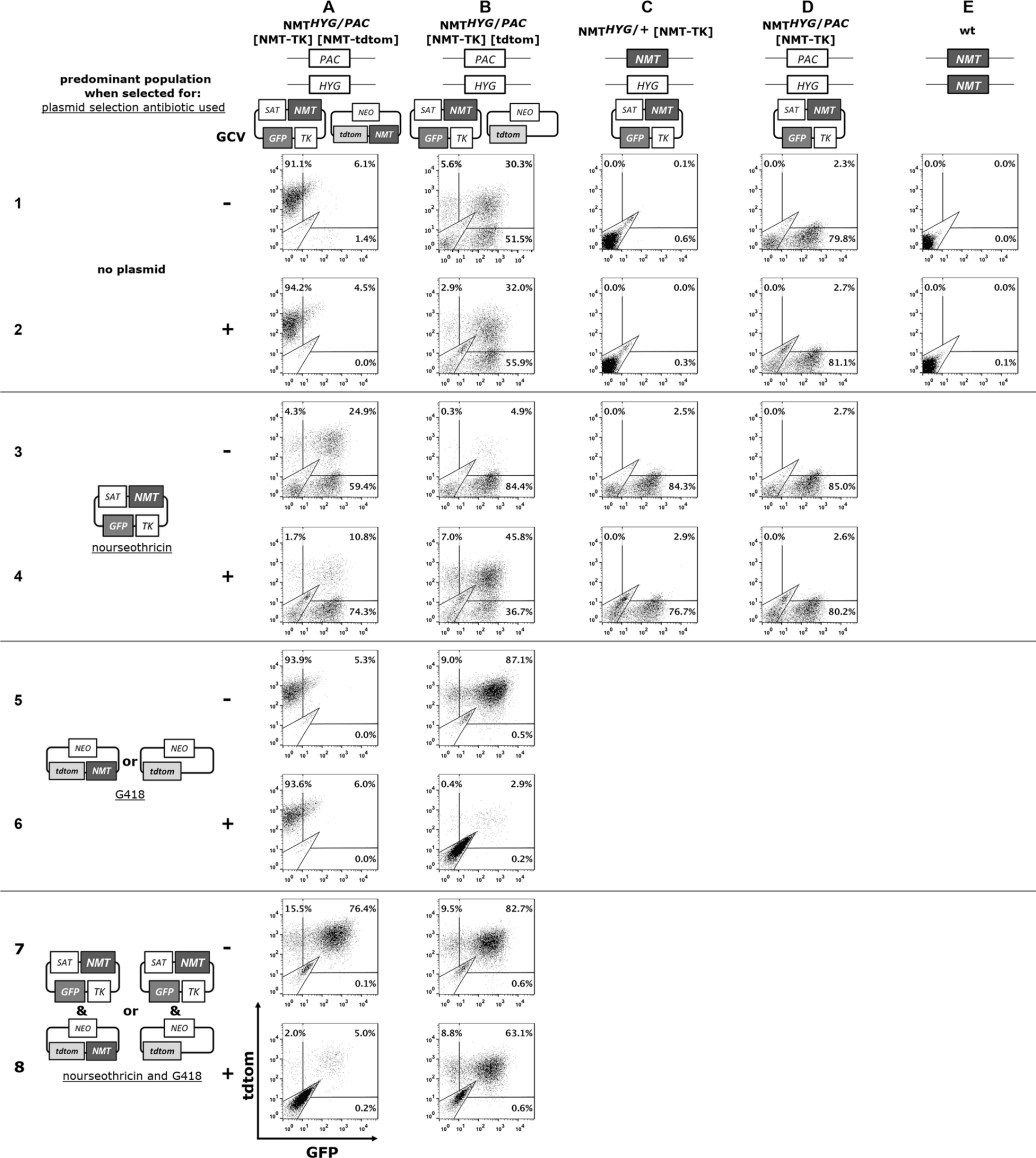
*NMT* is essential in *L. donovani* amastigotes: qualitative analysis. Representative flow cytometry plots (plotted y-axis: tdtom, x-axis: GFP) from parasites obtained from spleens of BALB/c mice treated for 28 days with 3 mg/kg per day− b.i.d with ganciclovir (+; GCV) or with solvent (−). The predominant population was identified for each condition (single or double knockout, transfected with NMT-TK plus either the NMT-tdtom or the tdtom plasmid) by selecting for no plasmid (medium only, wt; hygromycin *NMT*^*HYG*/+^ [NMT-TK]; all others hygromycin and puromycin) in the top two rows; for NMT-TK plasmid (as before plus nourseothricin; rows 3 and 4); for NMT-tdtom or tdtom plasmid (hygromycin, puromycin and G418; rows 5 and 6); for NMT-TK/NMT-tdtom or NMT-TK/tdtom plasmid (hygromycin, puromycin, nourseothricin and G418; rows 7 and 8). See Additional files 4, 5, 6, 7, 8: Figures S4–S8 for flow cytometry plots for each mouse. Flow cytometric analysis was performed 14 days after spleens were removed and parasites grew out from cell suspension

To investigate to what extent and under what circumstances the NMT-TK plasmid could be lost *in vivo*, groups of BALB/c mice were infected with stationary phase *L. donovani* promastigotes of the different transgenic lines as shown in Fig. 4. Mice were then treated twice daily with GCV starting at one week post-infection in order to make sure promastigotes have transformed into amastigotes and the infection had time to establish itself; Additional file 3: Figure S3 shows flow cytometry plots of the parasites used in these experiments. After a further 4 weeks, mice were sacrificed, and the spleens removed to determine the level of parasitaemia by limiting dilution assay (parasites are not cleared from the spleen over this time period; [37]). The extent to which parasites were resistant to the different combinations of antibiotics, and its correlation with retention of specific plasmids was also measured. Table 3 summarises the mutant lines of *L. donovani* used for the qualitative and quantitative plasmid retention experiments and the applied selection conditions. To further investigate the dynamics of plasmid retention, the same samples were also re-differentiated into promastigotes, grown with the respective antibiotics for single or double *NMT* KO s, and subsequently analysed by flow cytometry (Fig. 5). Each column represents a different mutant line and each row represents for which plasmid(s) was selected for by respective antibiotics, no plasmid i.e. total population, isolated from infected mice that were GCV-untreated (-) or GCV-treated (+) (Fig. 5, rows 1 and 2). In order to gain more insight how the population was composed, the parasite samples were subjected to the respective antibiotics to select for either plasmid or both together (Fig. 5, rows 3–8).

**Table 3:** *Leishmania donovani* mutant lines used for the qualitative and quantitative plasmid retention experiments in Figs. 5, 6, 7 and the applied selection conditions

Leishmania mutant line	Antibiotic used to select for specific plasmid(s)		
	nourseothricin	G418	nourseothricin/G418
LV9 *NMT*^*HYG*/+^ [NMT-TK]	NMT-TK	na	na
LV9 *NMT*^*HYG*/*PAC*^ [*NMT–TK*]	NMT-TK	na	na
LV9 *NMT*^*HYG*/*PAC*^ [*NMT–TK*] [tdtom]	NMT-TK	tdtom	NMT-TK; tdtom
LV9 *NMT*^*HYG*/*PAC*^ [*NMT–TK*] [*NMT*-tdtom]	NMT-TK	NMT-tdtom	NMT-TK; NMT-tdtom

*Abbreviation*: na, not applicable

The predominant population of splenic single *NMT* KOs had lost the NMT-TK plasmid (Fig. 5, column C, row 1 (C1 and C2) and Additional file 4: Figure S4), even in the untreated group. However, we were able to recover a small subset of parasites were this plasmid was still present, both treated or untreated as identified under appropriate selection with hygromycin and nourseothricin (Fig. 5, C3, C4). The NMT-TK plasmid, however, was retained in double *NMT* KOs under all selection conditions (Fig. 5, D1–D4; Additional file 5: Figure S5).

Double *NMT* KOs also possessing ectopic *NMT* alleles encoded by the NMT-TK or the NMT-tdtom plasmid were isolated from spleens of BALB/c mice. Both the GCV-treated and GCV-untreated parasites were exclusively tdtom^+^ (Fig. 5, A1, A2; Additional file 6: Figure S6), consistent with possession of only a single *NMT* allele. It was still possible to identify parasites as a GFP^+^ or as double positive (GFP^+^/tdtom^+^) population when selected for the NMT-TK plasmid (Fig. 5, A3, A4). No difference was observed in the qualitative analysis when these mutants were selected for the NMT-tdtom plasmid (Fig. 5, A5, A6). Both GCV-treated and GCV-untreated parasites resulted in a tdtom^+^ population, reflecting the result obtained in the majority of the population (Fig. 5, A1, A2). In GCV-untreated parasites, selection for NMT-TK and NMT-tdtom plasmids (Fig. 5, A7) resulted in tdtom+ and GFP^+^ populations, as in the original parasites injected. However, when we selected for parasites still carrying both plasmids, there were almost no double positive parasites left in the population extracted from mice treated with GCV (Fig. 5, A8). This result is in agreement with previous results (Fig. 5, C1–C4), confirming that the NMT-TK plasmid was lost while the NMT-tdtom plasmid was almost exclusively retained (i.e. Fig. 5, A2 and A6).

Double *NMT* KO parasites only possessing an ectopic *NMT* allele, encoded by the NMT-TK plasmid, when isolated from the spleens of GCV-treated and GCV-untreated BALB/c mice, were GFP^+^ or GFP*+*/tdtom^+^ in both groups (Fig. 5, B1 and B2; Additional file 7: Figure S7). Selecting for the NMT-TK plasmid (Fig. 5, B3, B4) in parasites isolated from untreated mice resulted in an almost exclusive GFP^+^ population. Similarly, selection for the NMT-TK plasmid in the GCV-treated group resulted in GFP^+^ (46%) and GFP^+^/tdtom^+^ (37%).

Selecting for the tdtom plasmid not encoding *NMT* in the GCV-untreated group (Fig. 5 B5) resulted in a mainly double positive (90% GFP^+^/tdtom^+^) population as in the original injected population. However, almost no parasites could be detected in the GCV-treated group and the few that were detected were also double positive (Fig. 5 B6). The double negative population was comprised of splenic cells as confirmed by microscopy.

In GCV-treated and GCV-untreated parasites, selection for the NMT-TK and tdtom plasmid (Fig. 5 B7, B8) resulted in GFP^+^/tdtom^+^ populations identical to the profile of the originally injected parasites.

In summary, the NMT-TK plasmid does not need to be maintained in the presence of another endogenous or ectopic gene copy of *NMT* even in the absence of negative drug pressure (Fig. 5, A1 and C1). In contrast, the NMT-TK plasmid is retained in the absence of another *NMT* gene (Fig. 5, B1 and D1).

The average median fluorescence intensity (MFI) values of the GFP fluorescence in the tdtom gate for the 4–5 animals are shown in Table 4. For the wt population the median MFI for GFP was determined by drawing a gate around the population and it was 2 and 2.1 in the GCV-untreated and GCV-treated population, respectively. The GFP MFI in the tdtom+ gate for double *NMT* KO [NMT-TK][tdtom] was 5.6 and double *NMT* KO [NMT-TK][NMT-tdtom] was 4.8 on the day of the injection. In the double *NMT* KO parasites possessing ectopic *NMT* alleles encoded by the NMT-TK or the NMT-tdtom plasmid in the GCV-treated groups the GFP MFI in the tdtom+ gate was very low (2.6 and 2.8, respectively) when only selected for the double KO and the red plasmid, respectively. Similarly the MFI values in the tdtom+ gate in the GCV-untreated groups, possessing only ectopic *NMT* alleles encoded by the NMT-TK or the NMT-tdtom plasmid and selection was only performed for the double KO or the red plasmid, were also very low (3.2 and 3.0, respectively). This confirms that these populations mainly or only possess the tdtom plasmid. In all other cases, the GFP MFI in the tdtom+ gate was between 4.0–4.4 as there was also a contribution of the NMT-TK plasmid with its GFP dim population.

**Table 4:** Average median fluorescence intensity (MFI) values of the GFP signal in the tdtom+ gate

Selection condition^a^	GCV	NMT ^HYG/PAC^ [NMT-TK] [tdtom]	NMT ^HYG/PAC^[NMT-TK] [NMT-tdtom]	wt
Entry^b^		5.6	4.8	
HP	−	3.6	3.2	
	+	4.7	2.6	
HPS	−	3.6	4.0	
	+	4.8	4.4	
HPN	−	4.3	3.0	
	+	5.3	2.8	
HPNS	−	5.0	4.4	
	+	6.2	4.0	
na	−			2.0
	+			2.1

^a^Antibiotic combinations used for group selection (H – Hygromycin, P – Puromycin, S – Nourseothricin, N – G418)

^b^MFI values of parasites at day of injection

*Abbreviation*: na, not applicable

In the double *NMT* KO parasites only possessing an ectopic *NMT* allele, encoded by the NMT-TK plasmid there was a clear skew towards higher MFI values (the lowest was 3.6 and the highest was 6.2). The lower values were only observed when only very few events were recorded in the tdtom+ gate. This confirms that there is always a contribution of the GFP dim population in the tdtom+ gate, highlighting the fact that the parasites need to maintain the NMT-TK plasmid.

### Quantitative *in vivo* analysis

**Fig. 6:**
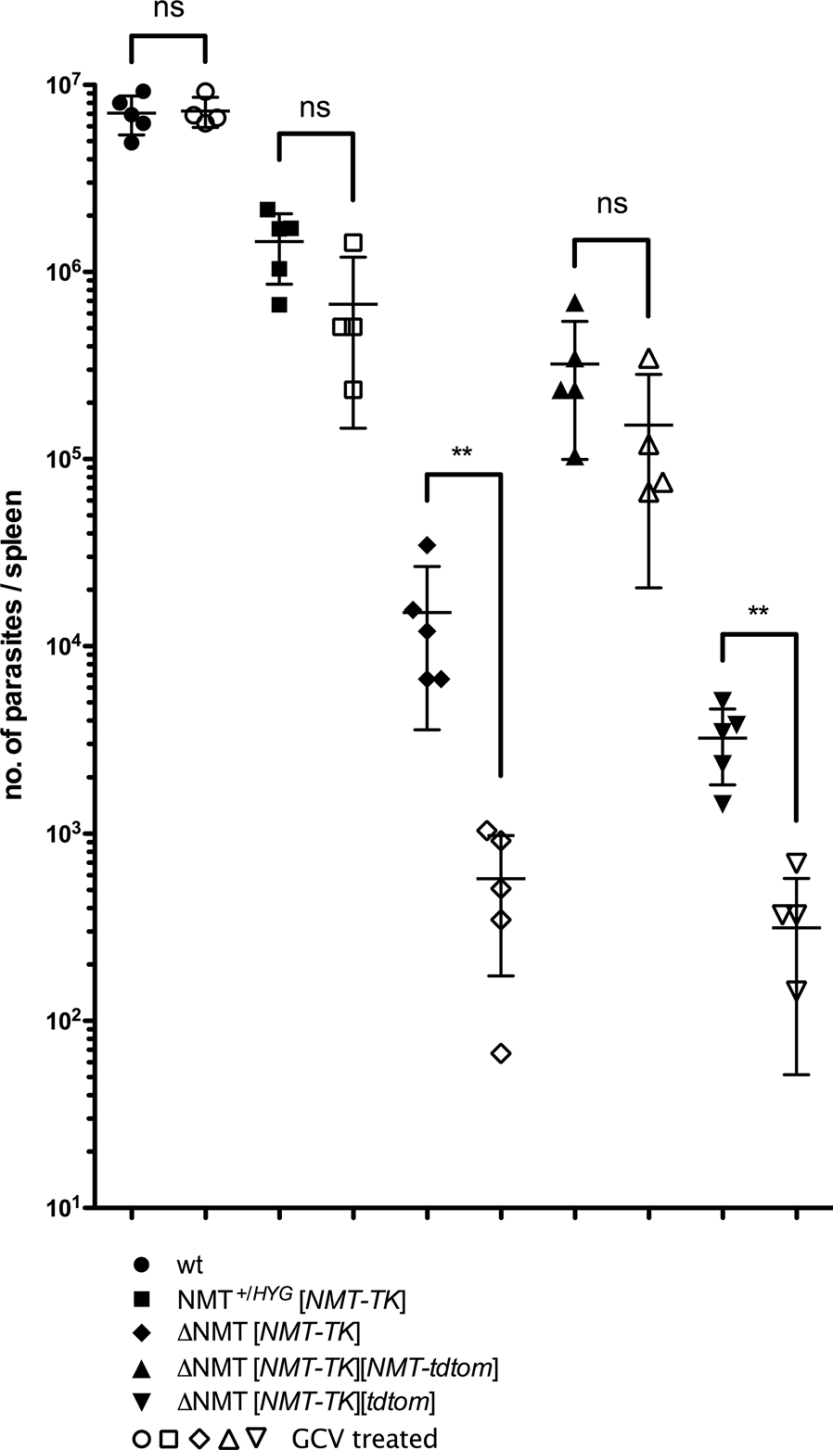
Quantitative analysis of total parasite burden. Parasite burden in spleen of BALB/c mice, 5 mice per group, after infection of spleen with LV9 wt, LV9 *NMT*^+/*HYG*^ [NMT-TK], LV9 Δ*NMT* [NMT-TK], LV9 Δ*NMT* [NMT-TK][NMT-tdtom] or LV9 Δ*NMT* [NMT-TK][tdtom] and treating with Ganciclovir (3 mg/kg per day; open symbols) or with diluent/solvent (filled symbols) for 28 days b.i.d. There was no difference in total parasite burden between treated and untreated wt parasites or when the second allele of *NMT* was present or *NMT* was ectopically expressed from a plasmid. However, the total parasite burden significantly dropped in mice upon treatment with GCV if parasites were double *NMT* KOs or it was not ectopically expressed from a plasmid. In the GCV-treated group of wt, LV9 *NMT*^+/*HYG*^ [NMT-TK] and LV9 Δ*NMT* [NMT-TK][NMT-tdtom], one animal had to be sacrificed for ethical reasons. Statistics: Mann–Whitney test *P < 0.5, **P < 0.05

The total burden of parasites in the spleen after 35 days of infection and 28 days of treatment was determined by limiting dilution assay (Fig. 6). There was no difference observed in the total splenic numbers of *L. donovani* wt parasites in solvent control or GCV-treated mice. No significant differences were observed in mice infected with single *NMT* KOs and expressing *NMT* from an ectopic locus nor if *NMT* was expressed solely ectopically from both plasmids LV9 *NMT*^+/*HYG*^ [NMT-TK] and LV9 Δ*NMT* [NMT-TK] [NMT-tdtom], although the parasite burdens were slightly less in the GCV-treated animals. However, parasite burdens were significantly reduced upon treatment with GCV in mice infected with *NMT* double KO parasites and with *NMT* only encoded by the NMT-TK plasmid (LV9 Δ*NMT* [NMT-TK] and LV9 Δ*NMT* [NMT-TK][tdtom]; Mann–Whitney U-test: *U* = 0, *P* = 0.0079).

All mutant lines resulted in a lower splenic parasite burden compared to the wt parasites. It was further noted that the parasite burdens were markedly reduced (Mann–Whitney U-test: *U* = 0, *P* = 0.0079) when *NMT* was only expressed from the NMT-TK plasmid as compared to the mutants in which *NMT* was also expressed from one genomic allele or from the NMT-tdtom plasmid, indicating that the correct regulation of *NMT* expression is important in this context. The burdens upon GCV treatment were comparable when *NMT* was only expressed from the NMT-TK plasmid (Fig. 6), although the burdens were lower in the solvent-treated mice when the parasites also carried the tdtom plasmid (Fig. 6).

The splenic parasite burden in the GCV untreated groups varied (Fig. 6). This suggested the over-expression of *NMT* and the regulation of its expression had an effect on the infectivity and/or the doubling-time of the parasites. Of note, this could be an effect due to the genetic manipulation in general and/or the general loss of infectivity observed when parasites are cultured axenically, a process particularly evident in *L. donovani* [38, 39]. Due to technical reasons, mutant lines were not passaged through mice as it was assumed that without selection for the plasmids by the respective antibiotics *in vivo*, dispensable plasmids would be lost rapidly; this assumption was later confirmed (see Fig. 5, A1, B1, C1 and D1).

**Fig. 7:**
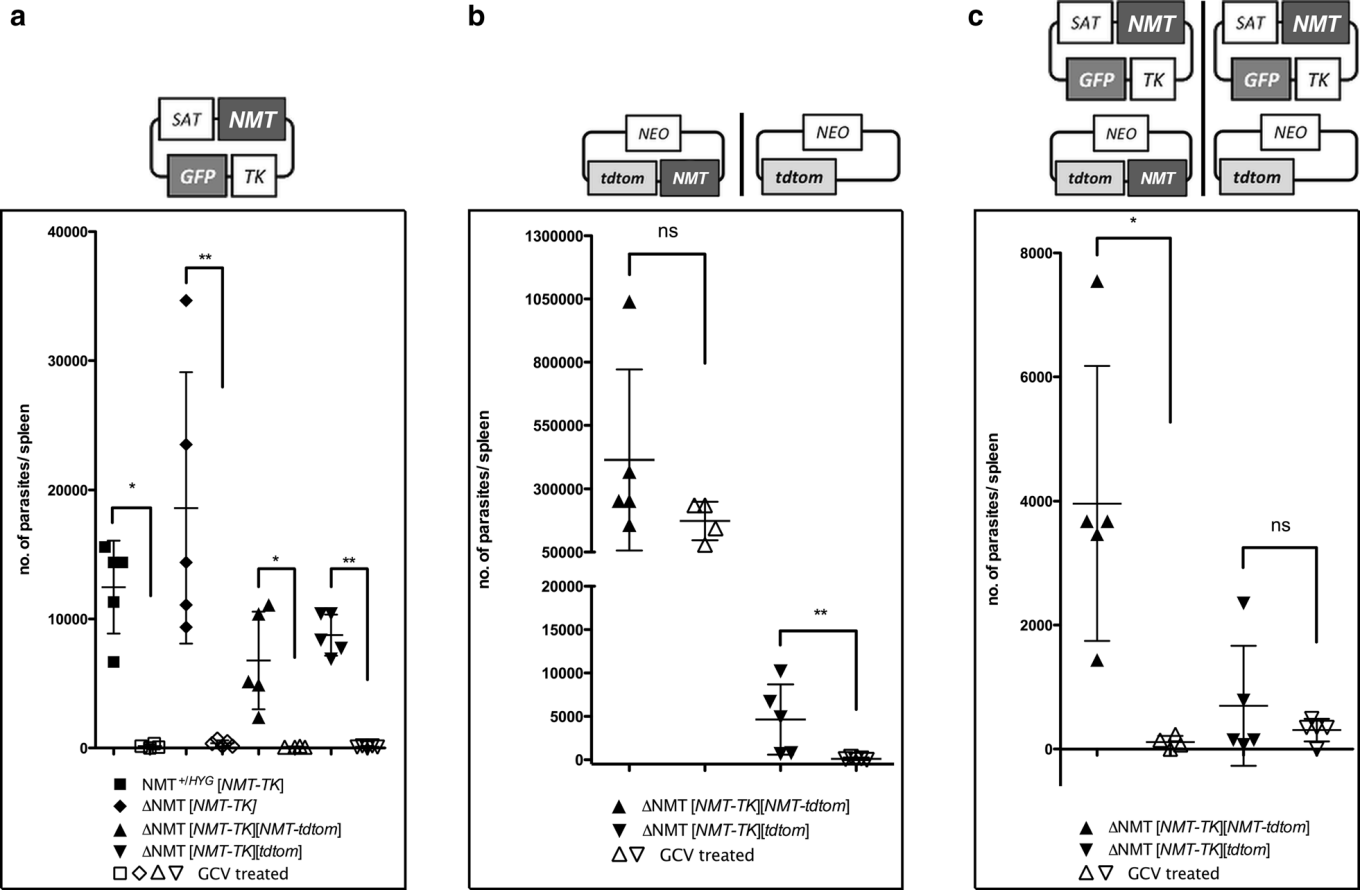
Quantitative analysis of the individual parasite burden and thus inferred plasmid retention. Proportion of parasite total burden in the spleen of BALB/c mice infected with LV9 *NMT*^+/*HYG*^ [NMT-TK], LV9 Δ*NMT* [NMT-TK], LV9 Δ*NMT* [NMT-TK][NMT-tdtom] or LV9 Δ*NMT* [NMT-TK][tdtom] are selected for by limiting dilution assay with the appropriate drugs. **a** Selection for the presence of plasmid coding for NMT, TK and GFP. Upon treatment with GCV for 28 days (3 mg/kg per day− b.i.d; open symbols), parasites have almost entirely lost the plasmid encoding for *TK*, *NMT, GFP.***b** Selection for the presence of the plasmid coding for NMT and tdtom or tdtom alone. Upon GCV treatment, there were ~100 times more parasites present if an additional *NMT* allele/gene was encoded. No significant difference between the untreated and treated group was observed. However, if no additional *NMT* allele/gene was present, parasites, survival was less (untreated group) and upon GCV treatment, those parasites were not detectable. **c** Selection for the presence of parasites positive for both plasmids, i.e. encoding NMT, TK & GFP and NMT & tdtom or only tdtom. Upon GCV treatment, only a few parasites were present in the spleen encoding for both plasmids. There were more parasites in the spleen in the group not encoding for a second *NMT* gene carrying both plasmids. However this number was not significant (Mann–Whitney test *P < 0.5, **P < 0.05)

In the surviving total splenic parasite population, it was further assessed as to what extent the plasmids were retained. This was also done by a limiting dilution assay but in the presence of the respective antibiotics (Fig. 7). Significantly lower burdens were observed upon GCV treatment compared to solvent treatment (Mann–Whitney U-test: *U* = 0, *P* = 0.0159 and Mann–Whitney U-test: *U* = 0, *P* = 0.0079), when subsequently selected for the NMT-TK plasmid. There were almost no parasites present in the spleen still carrying this NMT-TK plasmid (Fig. 7a). This was the case for all mutant lines in which *NMT* was present either as a genomic allele or expressed from an ectopic locus. On the other hand, a significant difference (Mann–Whitney U-test: *U* = 0, *P* = 0.0079) was observed in the splenic parasite burdens in mice upon treatment with GCV compared to the solvent-treated and subsequently selected for the tdtom plasmid (Fig. 7b). As expected, if NMT is essential and no genomic *NMT* allele is present, no significant difference in the parasite burden was observed when selected for the NMT-tdtom plasmid in the GCV or solvent-treated group (Fig. 7b), indicating the necessity for the presence of NMT for parasite viability.

In agreement with the hypothesis that *NMT* is an essential gene in amastigotes, a significant difference (Mann–Whitney U-test: *U* = 0, *P* = 0.0159) in the parasite burden was observed upon treatment with GCV and the subsequent selection for the NMT-TK and the NMT-tdtom plasmids as compared to the solvent group (Fig. 7c). This was due to loss of the NMT-TK plasmid. However, no difference was observed in the parasite burdens in mice treated with GCV or the solvent and subsequent selection for the NMT-TK and the tdtom plasmid (Fig. 7c), indicating that there is no selection in mice for the plasmid encoding only tdtom. This is in agreement with the qualitative data (Fig. 5, B1 and B2) where it was shown that the predominant population in the solvent-treated group had mostly lost the plasmid coding only for tdtom, while in the GCV-treated group the predominant population was only carrying the NMT-TK plasmid.

## Discussion

Determining the essentiality of a gene in *Leishmania* species, especially in intracellular amastigotes, is challenging. At present, an absolute requirement for parasite viability is assumed when it is only possible to fully delete a target gene of interest from its genomic locus when an ectopically expressed additional gene copy is present. This type of analysis has been exclusively performed in extracellular promastigote stages of the parasite life-cycle to date, with the information generated then correlated with similar function in intracellular amastigotes. Plasmid shuffle [31] provides an alternative approach for the positive discrimination of essential genes as it involves not only the deletion of the gene of interest from its genomic locus in the presence of an ectopic copy, but testing of whether this ectopic copy is dispensable upon negative selection [28–30]. Morales et al. [30] employed this approach to demonstrate an absolute requirement for the co-chaperone STI1 in parasite viability and its essential residues necessary for phosphorylation, while Dacher et al. [28] used similar methods to show that activity of the protein kinase LmaMPK4 is also required. However, both studies were carried out using extracellular promastigotes.

TK-expressing amastigotes of both *L. donovani* and *L. major* have already been described elsewhere. Muyombwe et al. [40] infected murine and human macrophages with *L. major* promastigotes expressing a TK plasmid and, following differentiation into amastigotes and subsequent treatment with GCV, showed decreased intracellular infection levels over time. Similarly, Ghedin et al. [41] expressed TK under the control of the A2 gene in *L. donovani* promastigotes, prior to infection of murine primary macrophages. Subsequent GCV treatment decreased infection levels substantially.

Here, we wanted to take the plasmid shuffle technique a step further and use it to prove the essentiality of a potential drug target gene in intracellular amastigotes. Using this approach, we first confirmed our previous observations that *NMT* is an essential gene in promastigotes in two *Leishmania* species [22, 23]. These experiments also demonstrated that the plasmid shuffle system was reproducible in our hands. We observed that during negative selection with GCV, a replicating population of parasites was necessary. Our data indicate that the TK-encoding plasmid is not actively lost but rather decreases in abundance through unequal division/propagation during mitosis and cytokinesis. Parasites without this plasmid then have a growth advantage and will outgrow the population which still contains the plasmid; as a result, 3–5 passages (i.e. ~25–50 generations) are needed to lose the plasmid *in vitro*. This also explains why we were unable to perform these experiments with different macrophage populations using the transgenic *L. donovani* parasites, which did not replicate fast enough intracellularly for detection *in vitro*. Other parasite species such as *Leishmania mexicana* or *L. amazonesis* could be more amenable to use of this *in vitro* strategy, as these strains replicate more rapidly in macrophage cell lines.

However, promastigotes expressing *NMT* only from the NMT-TK plasmid propagated even under GCV selection. This might be due to the inactivation of TK under negative selection as has been described in *T. brucei*, due to a single point mutation or a frame shift [42]. Although we cannot exclude that the GCV negative selection lead to TK inactivation here, it has been reported in previous *Leishmania* promastigote plasmid shuffle experiments that negative selection did not cause a similar TK mutation. Instead GCV was tolerated by the parasites [28]. Taken together, this indicates that *Leishmania* promastigotes can deal with GCV stress much better than *T. brucei*.

Given our focus on *L. donovani*, we therefore decided to work *in vivo* and chose a susceptible mouse strain that develops persistent splenic infection with a constant increase in splenic parasite burden over at least 112 days [37]. Mice were infected intravenously with promastigotes, with GCV treatment or control injections commencing 7 days later, after promastigote transformation and establishment of an amastigote infection. After 4 weeks of treatment, spleen parasite burdens and compositions were determined, setting up limiting dilution assays with the respective antibiotics to select for parasites still carrying both or either plasmid. The quantitative analysis revealed that NMT is essential for viability in intracellular amastigotes of *L. donovani*, hence confirming the validity of this enzyme as a target for drug development. The data also showed that a plasmid coding for an essential gene and TK can only be lost *in vitro* when a functional copy of the essential gene is present, even in the presence of GCV. *In vivo*, on the other hand, it appears that these parasites die either by necrosis or immune clearance. However, clearance of all parasites from spleens was not complete as promastigotes for flow cytometric analysis grew out in 8 out of 9 animals if *NMT* was only encoded by an episome. This highlights the fact that some parasites might undergo quiescence [43] upon negative selection pressure and replication of parasites resumes once negative selection pressure is removed. This circumstance could lead to recrudescence in a chemical intervention. It is also possible that the treatment has just not been long enough as no promastigotes grew out of the spleen for one animal indicating complete clearance is possible. The qualitative analysis appears to indicate that the GCV treatment *in vivo* is not necessary, as there is no apparent difference between GCV-treated and untreated. However, these discrepancies come from the fact that the flow cytometric analysis was done some time after the parasites have been freed from the spleens, thus what was present at the end of the GCV treatment was then again amplified. There was a 10-fold difference in the quantitative analysis in the amount of parasites present in the spleen between the treated and untreated group if *NMT* is only encoded on a single chromosome or plasmid. Quantitative PCR on the day of parasite isolation could be more precise in determining the plasmid levels present in the parasite population. However, the NMT-TK plasmid appears to have an intrinsic toxicity as it is not maintained over the period of the experiments in the GCV-untreated single *NMT* KOs group or in the double *NMT* KOs if the NMT-tdtom plasmid is present. In contrast, in the *NMT* KOs the NMT-TK plasmid is maintained if it is the sole source for *NMT* expression. Possibly the use of almost identical plasmids only differing by the fluorescent marker and encoding for an inactivated TK will reduce the inherent negative selection pressure due to varying expression levels of the gene of interest, the size of the plasmids, and replication efficiency of plasmids. The data also show that analysis of the plasmid retention in the total population confirms the essentiality of NMT sufficiently and the subsequent dissection of the respective retention is not necessary.

## Conclusions

Taken together, the results support that NMT is essential *in vivo*. As demonstrated here and in the absence of a robust inducible expression system, plasmid shuffle is a useful and powerful tool to validate the essentiality of a gene of interest in an intracellular pathogen *in vivo* and hence robustly confirm its suitability as a therapeutic target. This approach could also be used to characterize a gene product both temporally and functionally in order to, e.g. determine whether an active phosphorylation site is required for establishment of infection or for longer-term intracellular survival, or to what extent protein domains are utilized in infective amastigotes as compared to vector-transmitted promastigotes of *Leishmania.* Overall, these *in vitro* and *in vivo* data are consistent with the hypothesis that *NMT* is an essential gene for parasite viability and show, to our knowledge for the first time, that this target gene is not only essential in extracellular promastigotes but also in the intracellular amastigotes of *L. donovani*, the causative agent of human visceral leishmaniasis.

## Supplementary information

**Additional file 1: Figure S1.** Immunoblot analysis of *NMT* complemented double replacements.

**Additional file 2: Figure S2.** Flow cytometry characteristics of *L. donovani* GFP-dim promastigotes.

**Additional file 3: Figure S3.** Flow cytometry characteristics of *L. donovani* promastigote mutants on day of injection.

**Additional file 4: Figure S4.** Qualitative analysis of *L. donovani NMT*^*HYG*/+^ [NMT-TK] obtained from spleens of infected mice.

**Additional file 5: Figure S5.** Qualitative analysis of *L. donovani NMT*^*HYG*/*PAC*^ [NMT-TK] obtained from spleens of infected mice.

**Additional file 6: Figure S6.** Qualitative analysis of *L. donovani NMT*^*HYG*/*PAC*^ [NMT-TK][NMT-tdtom] obtained from spleens of infected mice.

**Additional file 7: Figure S7.** Qualitative analysis of *L. donovani NMT*^*HYG*/*PAC*^ [NMT-TK][tdtom] obtained from spleens of infected mice.

**Additional file 8: Figure S8.** Qualitative analysis of wild type *L. donovani* obtained from spleens of infected mice.

## Data Availability

Data supporting the conclusions of this article are included within the article and its additional files. Raw data are available from the corresponding author upon request.

## Abbreviations

b.i.d: bis in die (twice a day)
GCV: ganciclovir
ess.: essential
GFP: green fluorescent protein
HYG: hygromycin phosphotransferase
IR: intergenic region
KO: knockout
MFI: median fluorescence intensity
NEO: neomycin phosphotransferase
NMT: *N*-myristoyltransferase
non-ess.: non-essential
ORF: open reading frame
PAC: puromycin *N*-acetyltransferase
PMA: phorbol 12-myristate 13-acetate
SAT: S-streptothricin acetyltransferase
tdtom: tandem tomato fluorescent protein (*tdTomato*)
TK: thymidine kinase
