# Supplementary figures and images for: Genetic validation of Leishmania genes essential for amastigote survival in vivo using N-myristoyltransferase as a model
Source: Parasit Vectors. 2020 Mar 14;13:132. doi: 10.1186/s13071-020-3999-1 (PMC7071782; doi:10.1186/s13071-020-3999-1)

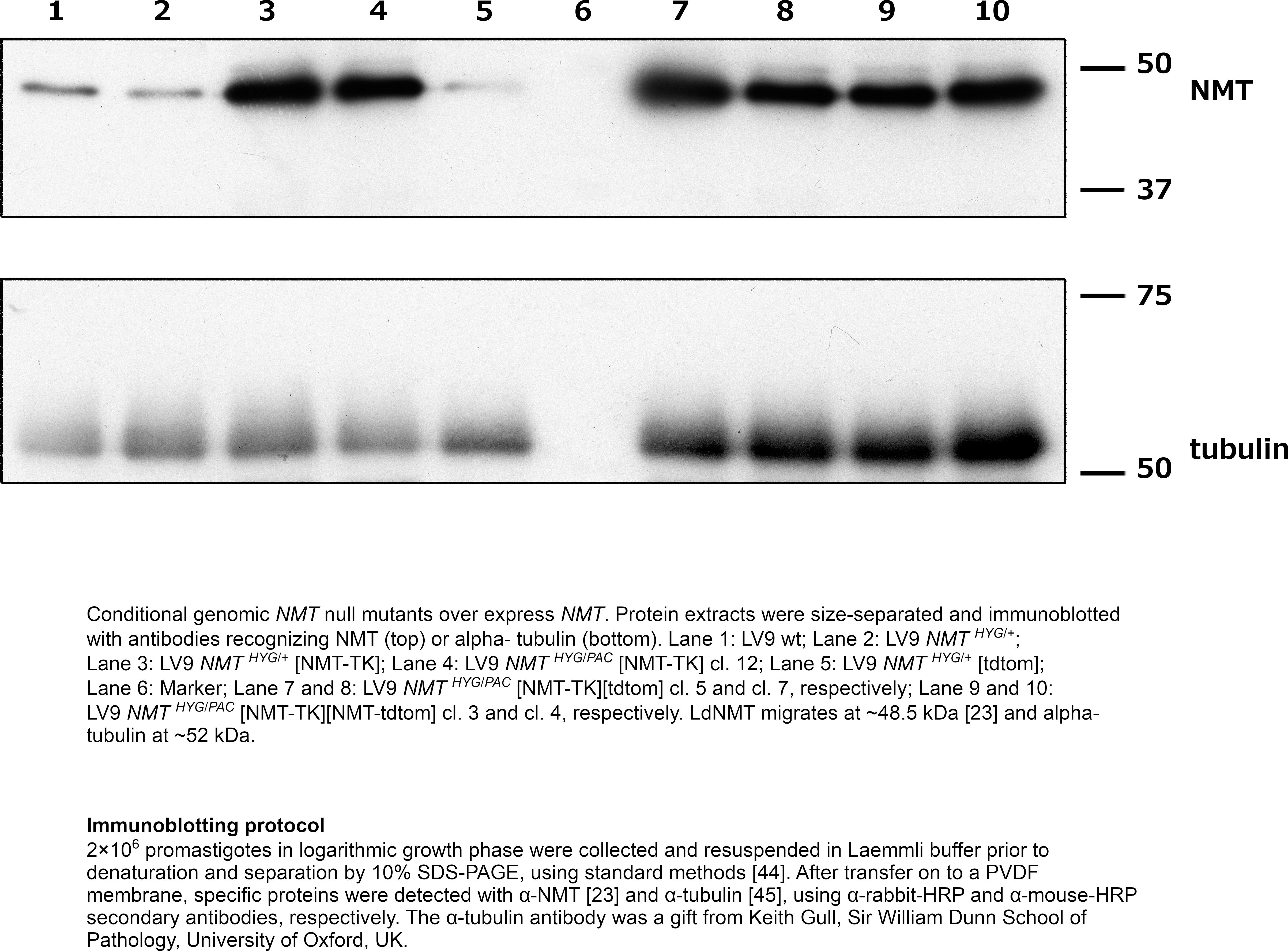

Supplement: Supplementary file 1 — Additional file 1: Figure S1. Immunoblot analysis of NMT complemented double replacements. [file 13071_2020_3999_MOESM1_ESM.tif]

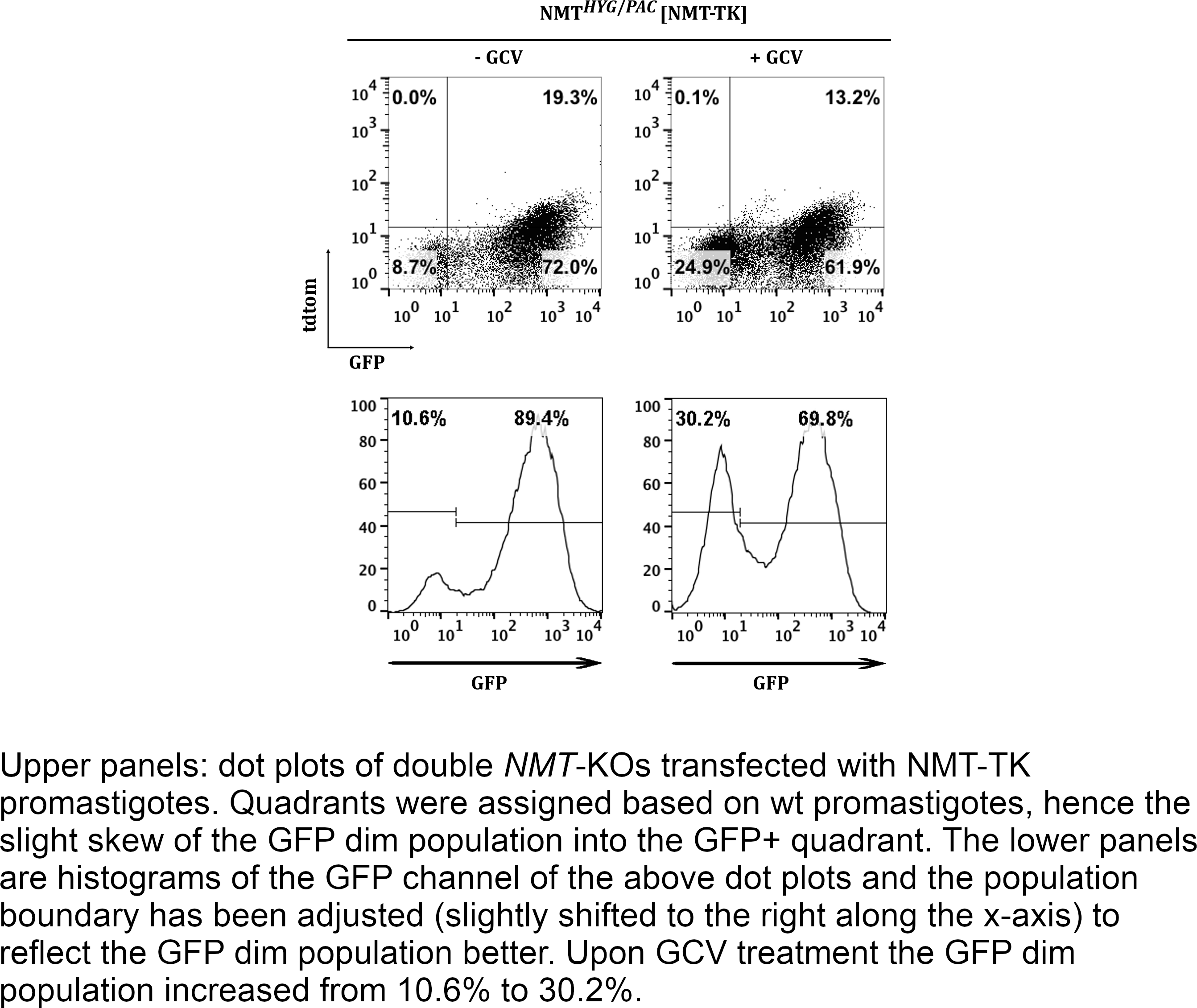

Supplement: Supplementary file 2 — Additional file 2: Figure S2. Flow cytometry characteristics of L. donovani GFP-dim promastigotes. [file 13071_2020_3999_MOESM2_ESM.tif]

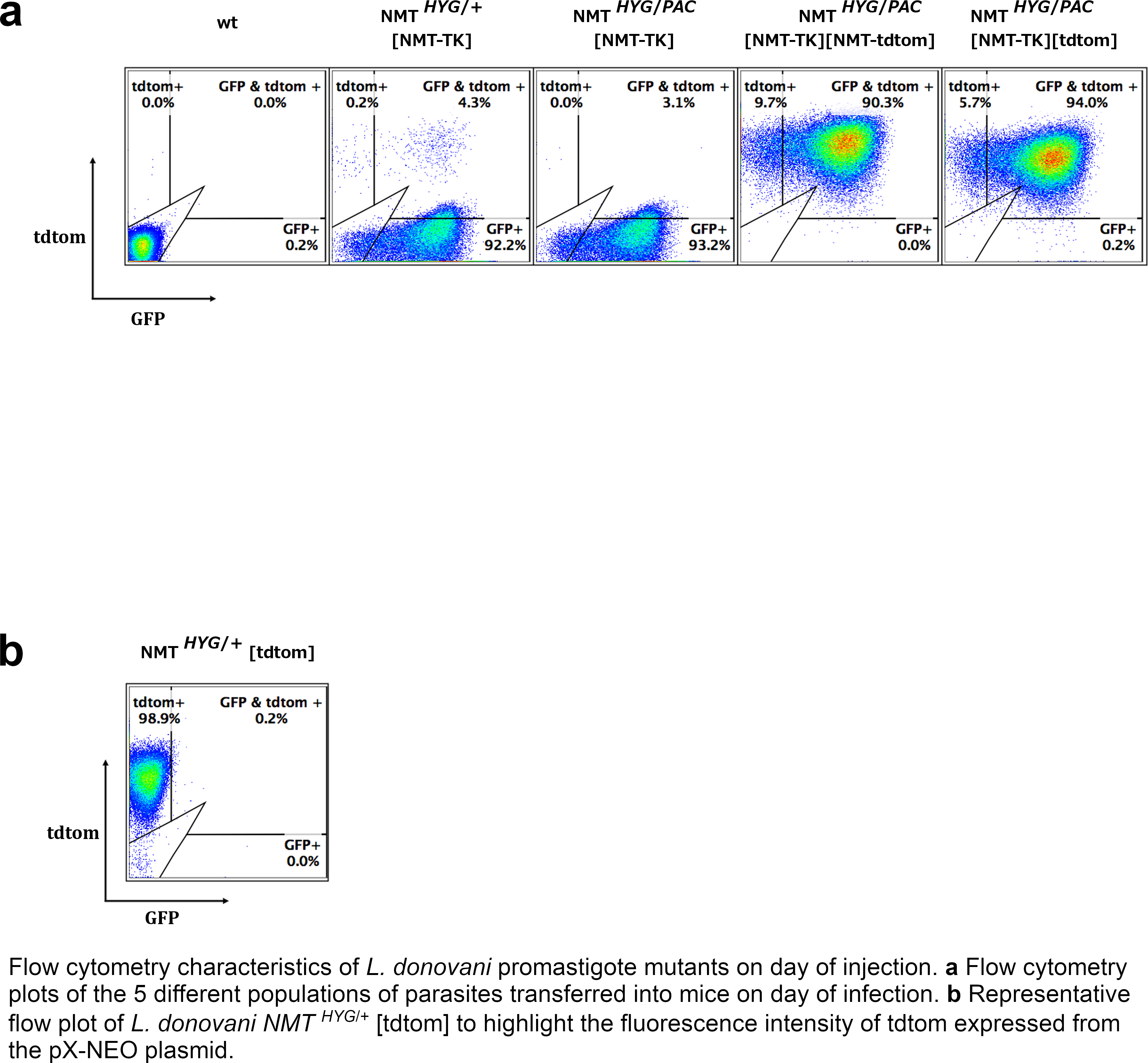

Supplement: Supplementary file 3 — Additional file 3: Figure S3. Flow cytometry characteristics of L. donovani promastigote mutants on day of injection. [file 13071_2020_3999_MOESM3_ESM.tif]

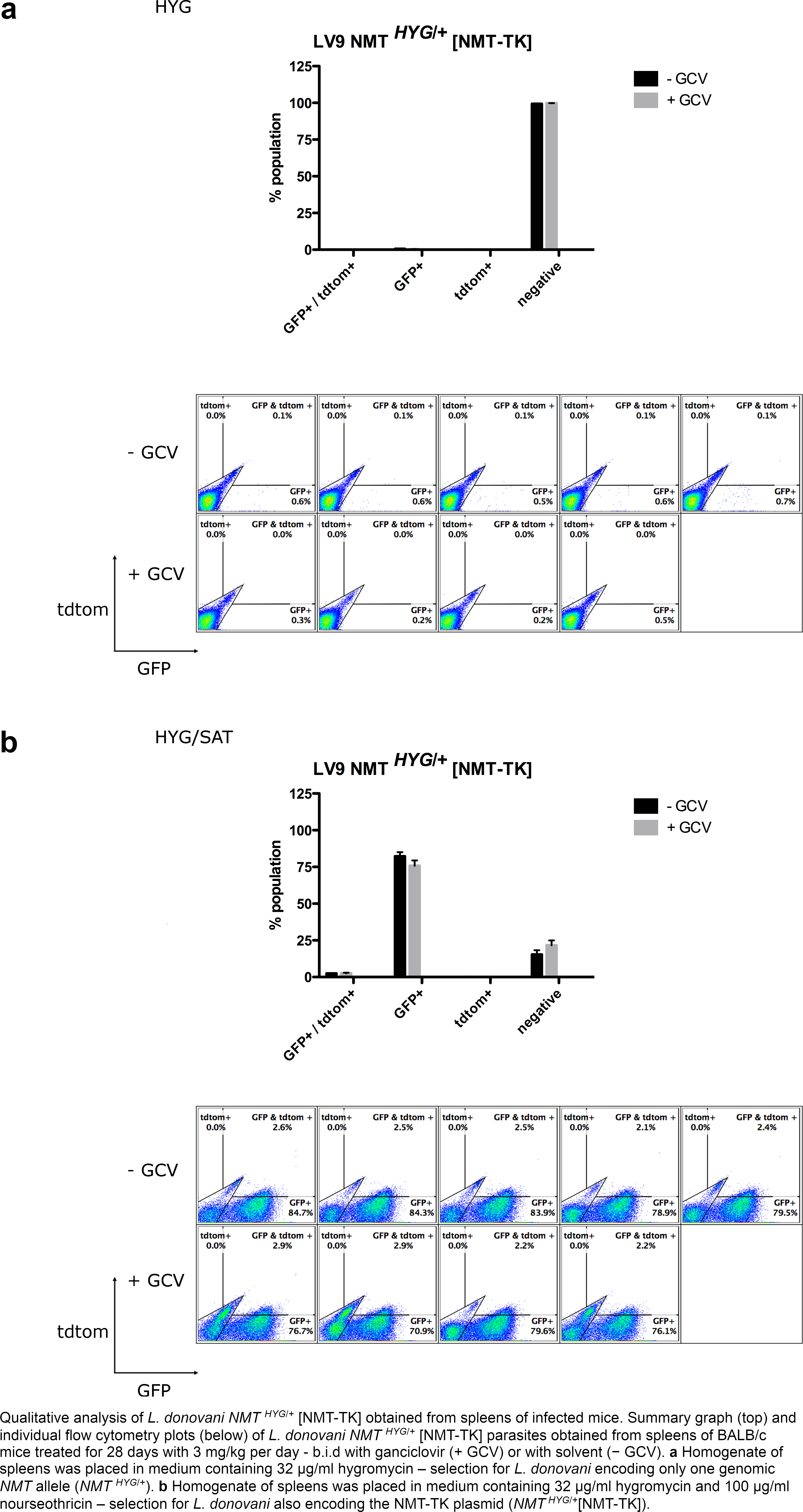

Supplement: Supplementary file 4 — Additional file 4: Figure S4. Qualitative analysis of L. donovani NMTHYG/+ [NMT-TK] obtained from spleens of infected mice. [file 13071_2020_3999_MOESM4_ESM.tif]

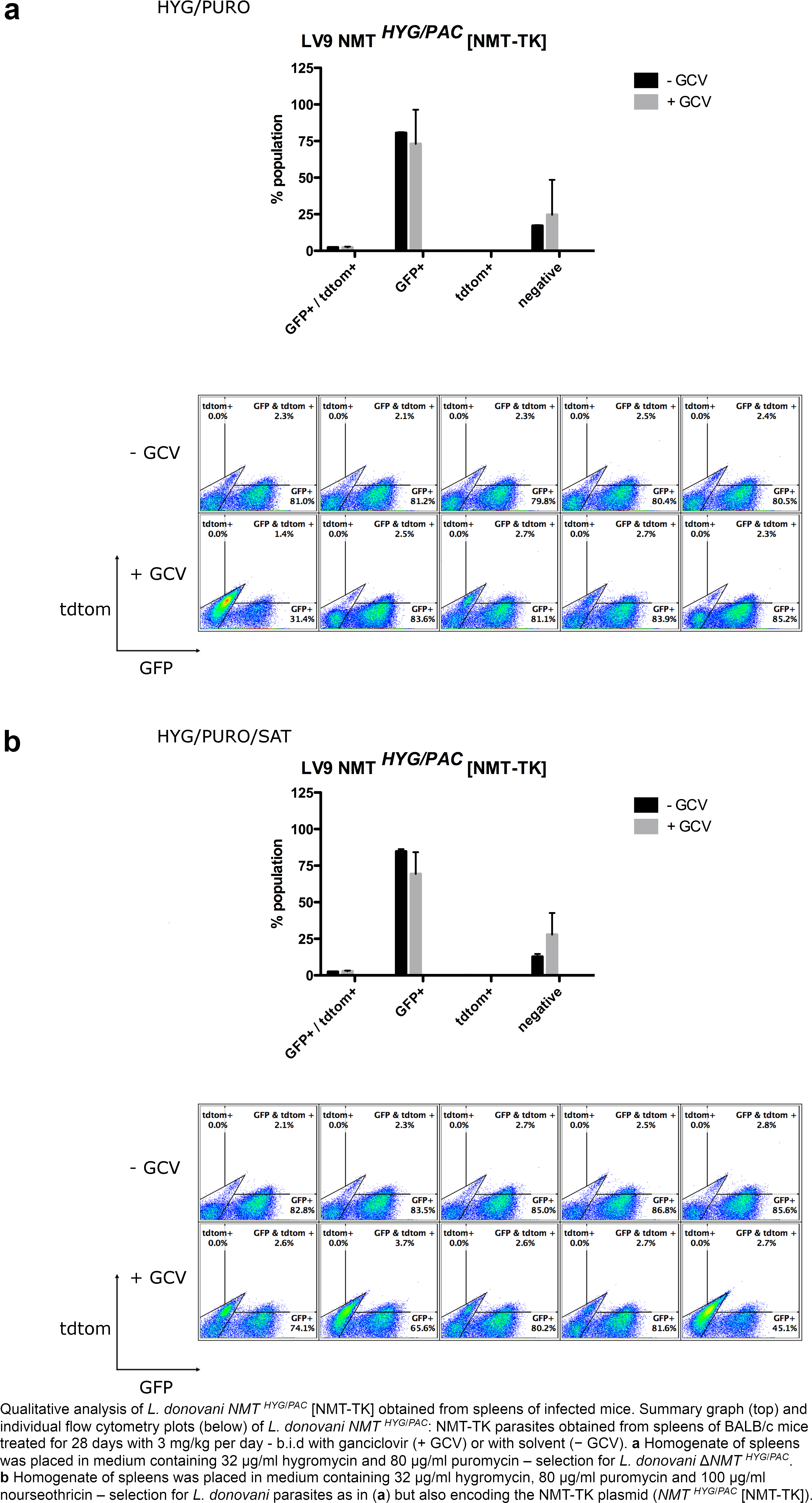

Supplement: Supplementary file 5 — Additional file 5: Figure S5. Qualitative analysis of L. donovani NMTHYG/PAC [NMT-TK] obtained from spleens of infected mice. [file 13071_2020_3999_MOESM5_ESM.tif]

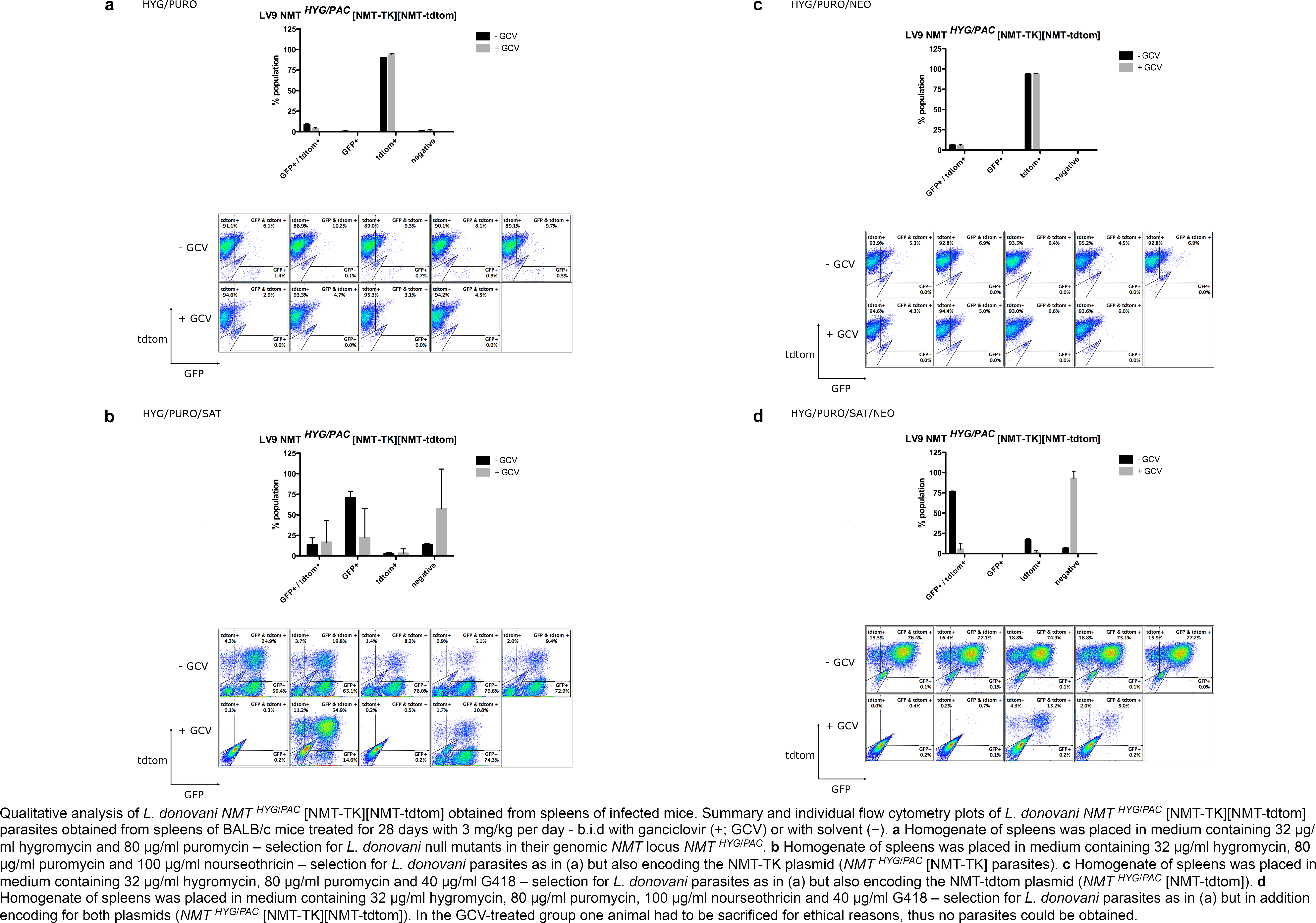

Supplement: Supplementary file 6 — Additional file 6: Figure S6. Qualitative analysis of L. donovani NMTHYG/PAC [NMT-TK][NMT-tdtom] obtained from spleens of infected mice. [file 13071_2020_3999_MOESM6_ESM.tif]

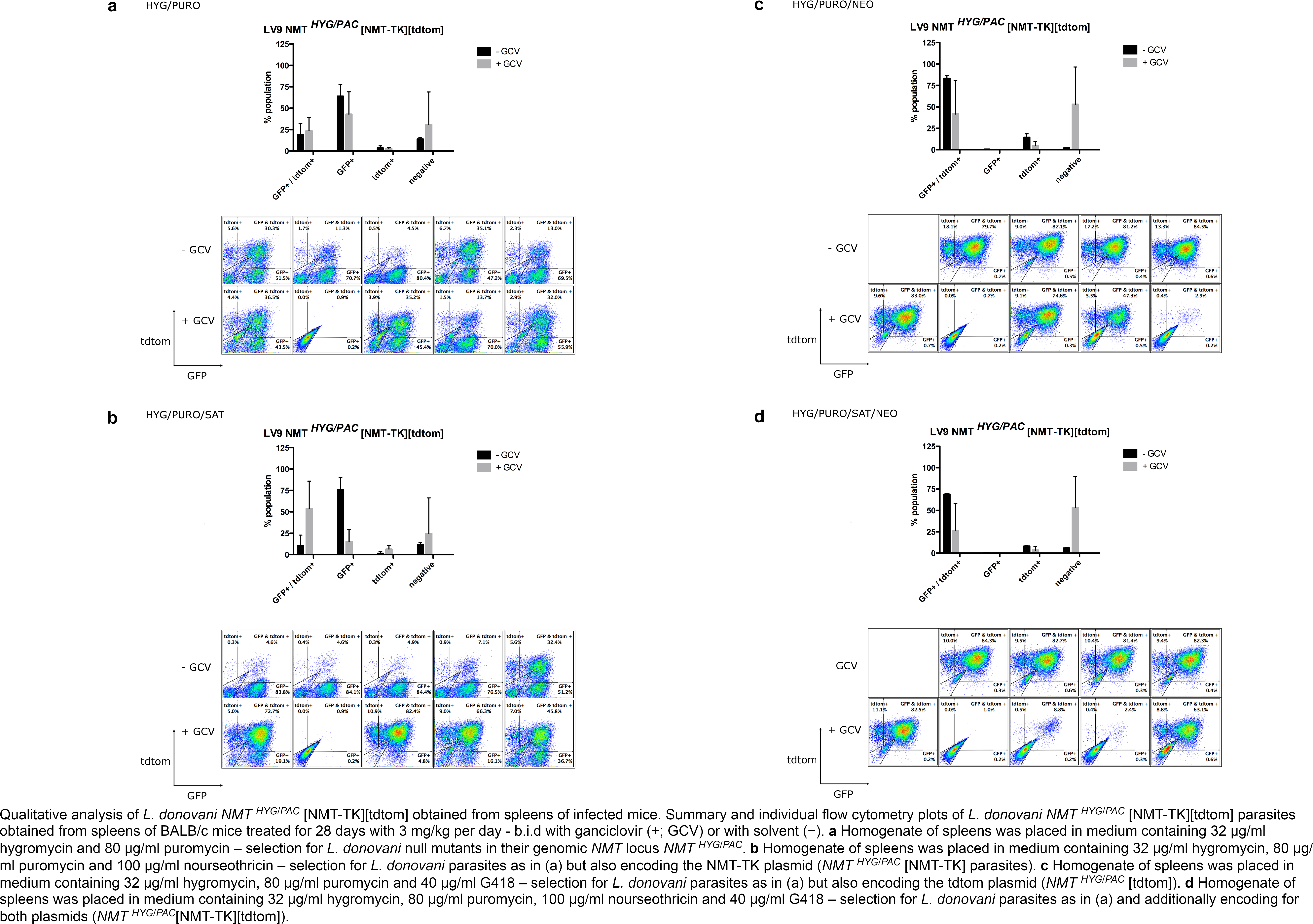

Supplement: Supplementary file 7 — Additional file 7: Figure S7. Qualitative analysis of L. donovani NMTHYG/PAC [NMT-TK][tdtom] obtained from spleens of infected mice. [file 13071_2020_3999_MOESM7_ESM.tif]

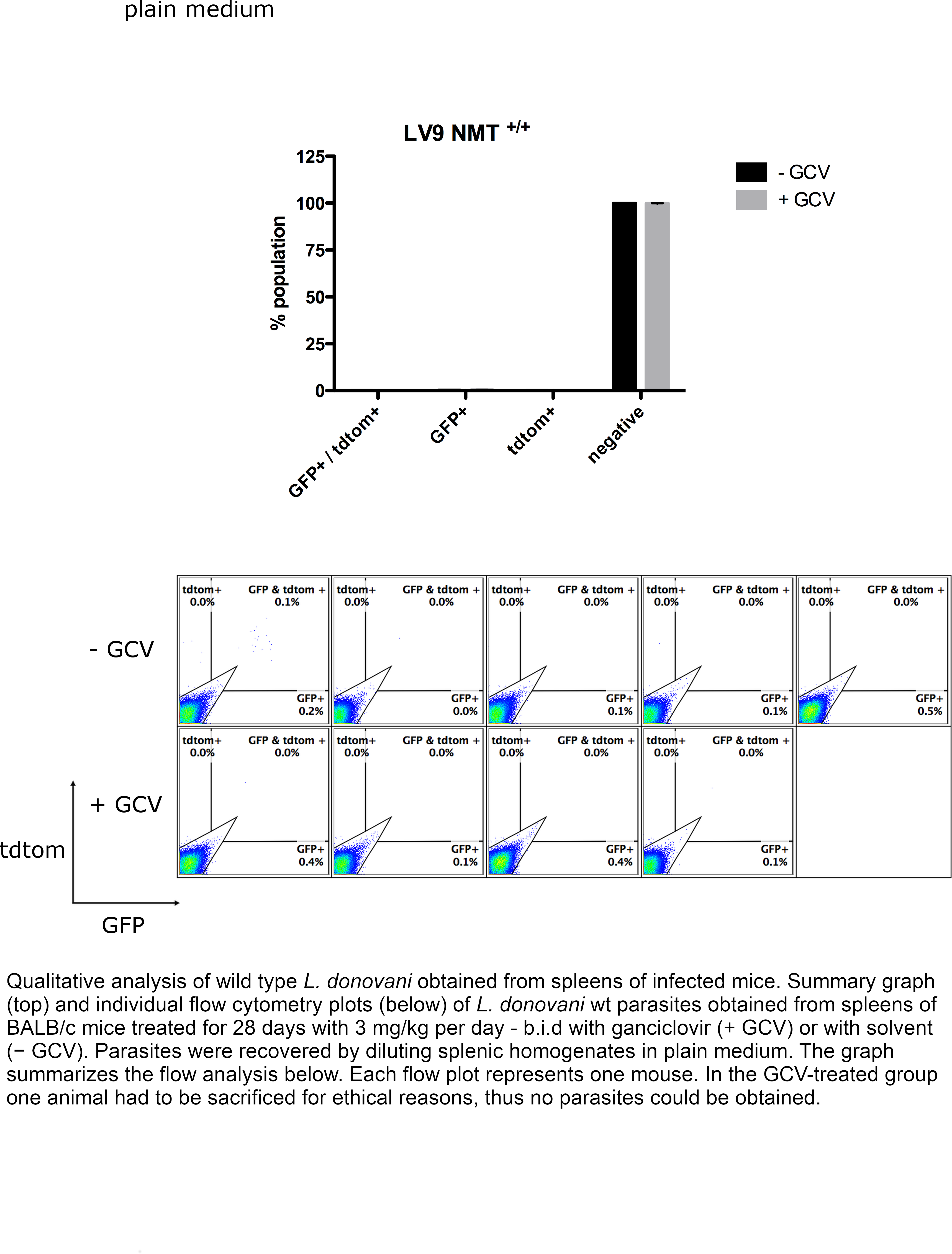

Supplement: Supplementary file 8 — Additional file 8: Figure S8. Qualitative analysis of wild type L. donovani obtained from spleens of infected mice. [file 13071_2020_3999_MOESM8_ESM.tif]
